# Impairment of stromal-epithelial regenerative cross-talk in Hirschsprung disease primes for the progression to enterocolitis

**DOI:** 10.1126/scitranslmed.adp4679

**Published:** 2025-07-30

**Authors:** Zhen Zhang, Dorothy Lee, Lingya Liu, Yi Xiong, Carol Lee, Ji-Eun Kim, Sinobol Chusilp, Ethan Lau, Yina Tian, Mehrsa Feizi, Mashriq Alganabi, Anthea Lafreniere, Tianran Cheng, Ruijie Zhou, Lu Han, Lihua Wu, Ping Xiao, Ya Gao, Giada Benedetti, Lucy Holland, Lucinda Tullie, Giovanni Giuseppe Giobbe, Long Li, Qi Li, Atsuyuki Yamataka, Vivian SW Li, Paolo De Coppi, Qian Jiang, Agostino Pierro, Bo Li

**Affiliations:** 1 Department of General Surgery, Capital Institute of Pediatrics Affiliated Children’s Hospital; Beijing, 100020, China; 2 Department of Translational Medicine, Division of General and Thoracic Surgery, The Hospital for Sick Children; Toronto, ON M5G 1X8, Canada; 3 Department of Physiology, University of Toronto; Toronto, ON, M5S 1A1, Canada; 4 Department of Medical Genetics, Capital Institute of Pediatrics; Beijing, 100020, China; 5 Xiangya School of Medicine, Central South University; Changsha, Hunan, 410008, China; 6 Department of Experimental Animal Research, Biomedical Research Institute, Seoul National University Hospital; Seoul, 08826, Korea; 7 Department of Pathology, The Hospital for Sick Children; Toronto, ON, M5G 1X8, Canada; 8 Department of Pathology, Capital Institute of Pediatrics Affiliated Children’s Hospital; Beijing 100020, China; 9 Stem Cell and Regenerative Medicine Section, Great Ormond Street Institute of Child Health, University College London; London, WC1N 1EH, United Kingdom; 10 Stem Cell and Cancer Biology Laboratory, The Francis Crick Institute; London, NW1 1AT, United Kingdom; 11 Department of Pediatric General and Urogenital Surgery, Juntendo University School of Medicine; Tokyo, 1138421, Japan; 12 Department of Specialist Neonatal and Paediatric Surgery, Great Ormond Street Hospital; London, UK; 13 Institute of Basic Medicine, Chinese Academy of Medical Sciences & School of Basic Medicine, Peking Union Medical College; Beijing, 100005, China; 14 Research Unit of Minimally Invasive Pediatric Surgery on Diagnosis and Treatment (2021RU015), Chinese Academy of Medical Sciences; Beijing, 100005, China

## Abstract

Hirschsprung’s disease (HSCR) is a congenital condition characterized by the improper migration of enteric neural crest cells, leading to bowel aganglionosis. This severe and life-threatening disorder often results in the development of Hirschsprung-associated enterocolitis (HAEC), which can occur before or after surgical resection of the affected bowel segment. Using human colonic tissue from HSCR patients alongside a well-established mouse model, we investigated epithelial regeneration dynamics and stromal-epithelium crosstalk in the distal ganglionic colon, a critical site for HAEC development. Our findings reveal that, in individuals with HSCR but without epithelial damage, the distal ganglionic colon displayed impaired epithelial regeneration and alteration of intestinal stem cell (ISC) dynamics, characterized by the induction of Clu+ ISC presence. This impaired regeneration ability precedes HAEC when epithelial damage occurs on-site. HSCR patients also exhibit remodeling in stromal cells in this distal ganglionic colon region, with fewer primary sources of WNT signal-releasing stromal cells (Stromal 2 cells) and the exclusive presence of inflammatory stromal cells (MMP1+ Stromal 4 cells). Stromal cells from the HSCR ganglionic colon failed to sustain the growth of colonic organoids derived from healthy controls; however, ibuprofen suppressed the pro-inflammatory subtype of stromal cells from the distal colon of HSCR patients, leading to effective restoration of epithelial organoid growth. These insights underscore the crucial role of impaired stromal-epithelium crosstalk in HSCR complications, highlighting HAEC pathogenesis and potential therapeutic targets. These observations on epithelial-stromal crosstalk dynamics could significantly impact the management and treatment of intestinal disorders in affected children, potentially improving outcomes and quality of life.

## Graphical abstract

**Graphic summary:**
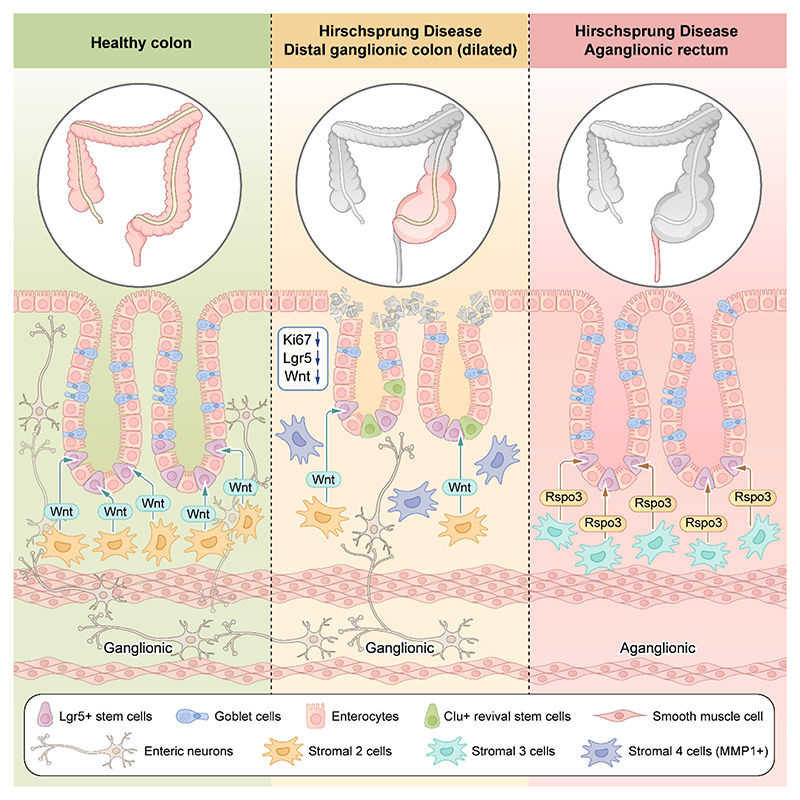

## Introduction

Hirschsprung disease (HSCR) is a congenital disease characterized by the absence of intrinsic ganglion cells in the submucosal and myenteric plexuses layers of the distal colon (1) and can lead to intestinal obstruction in neonates and infants (2, 3). This obstruction impedes the normal passage of stool through the affected aganglionic colon segments, leading to adjacent colon distention and the development of Hirschsprung-associated enterocolitis (HAEC), a life-threatening complication of HSCR (4). While surgical resection of the aganglionic bowel is the current standard surgical intervention aimed at relieving bowel obstruction, HAEC can also occur after the corrective surgery (5). While current research in this field has focused on understanding the impairment of migration of neural crest cells and their potential replacement to avoid resection of the aganglionic colonic segments, epithelial homeostasis in HSCR has not been fully investigated, and therefore HAEC pathophysiology remains poorly understood.

The intestinal epithelium has a remarkable capacity to undergo self-renewal and regeneration to maintain homeostasis (6). Intestinal stem cells (ISC) residing in the intestinal crypts are marked by the Leucine-rich repeat-containing G-protein coupled receptor Lgr5, and serve as the origin of all intestinal epithelial cells (7). Newborn cells migrate from the crypt along the crypt-villus axis, and acquire specific functions as they differentiate into distinct epithelial cell lineages (8). During intestinal injury, this balance shifts towards regenerating more newborn cells to replace or repair the intestinal epithelium and avoiding further damage (9). However, in cases of severe or prolonged injury, or when the regenerative capacity of LGR5-positive cells is compromised, +4 cells—reserve intestinal stem cells (+4 RSC) positioned above the base of the crypt—can be activated to replenish the stem cell pool (10–12). Recently, a small subset of Clu+ “revival stem cells” or revSCs has been reported, driving regeneration by replenishing Lgr5+ ISCs lost during injury (13). The involvement of epithelial regeneration and ISC dynamics during the initiation and progression of HAEC remains unexplored.

Wnt signaling has recently emerged as a critical pathway in maintaining the epithelial stem cell niche and its regenerative ability. Indeed, Wnt signaling directly regulates LGR5-positive ISCs, initiating a signaling cascade that promotes their self-renewal and proliferation, which plays a crucial role in ISC maintenance and homeostasis (14–16). It has been shown that there are redundant sources of Wnt within the intestine, including epithelial Paneth cells (17, 18) and stromal cells located underneath the epithelium (19, 20), as well as submucosal immune cell such as macrophages (21, 22). The balance between the different sources of Wnt signalling is critical for maintaining intestinal equilibrium, bolstering stem cell function, and promoting tissue repair, all of which have implications for disease progression (23, 24); however, the absence of Paneth cells in the colon poses a challenge to the understanding of Wnt signaling sources for epithelial regeneration during HAEC injury.

Intestinal stromal cells are located underneath the epithelium and secrete growth factors, such as Wnt and R-spondin, that are crucial for maintaining the self-renewal capacity of the stem cells (20, 25). Additionally, stromal cells play a pivotal role in creating a three-dimensional scaffold of the intestinal structure by producing extracellular matrix components like collagen, which is crucial for tissue stiffness and contributes to fibrosis by the excessive accumulation of scar tissue in the intestinal wall (26). Recent findings have indicated the development of fibrosis in the aganglionic colon of HSCR (27), leading to stricture formation. Remodeling of colon stromal cells has been well studied in the injury formation and epithelial regeneration in inflammatory bowel disease (IBD) (19, 28); however, the stromal-epithelial crosstalk in the pathogenesis of HAEC remains unknown.

Recently, researchers have explored strategies to manipulate the stromal cell and stem cell niche for therapeutic purposes to offset intestinal injury. This includes potential approaches to promote tissue repair, enhance the efficacy of stem cell-based therapies, and intervene in diseases that involve the intestinal epithelium. While the path to understanding HSCR and its progression to HAEC remains challenging due to limited clarity on the disease’s early stages, recent advancements in research models offer promising avenues for exploration. The Endothelin receptor B (*Ednrb*) mutant mouse is a well-described model of colorectal aganglionosis and a pertinent model for studying the proceeding transition from HSCR to HAEC. Indeed, the *Ednrb* mutant mouse model exhibits many features similar to human HSCR, including megacolon (29, 30), as well as alterations in the colonic microbiome, goblet cell function, immune regulation, and mucosal structure (31–33). Complementary work from intestinal organoids derived from purified crypt cells further aids investigations of disease progression and screening of potential therapeutic agents (34–36).

Building on the insights gained from human intestinal single-cell RNA sequencing (scRNA-seq) studies regarding enteric nervous system development and its role in disease in infants and children (37), recent research revealed the dynamic gene expression patterns along the differentiation and spatial axis of the digestive tract (38). Studies have also provided valuable information on the diversity and complex interactions of intestinal cell types, particularly enteric neural cell lineages, and their association with HSCR (39). Despite these advancements, knowledge gaps remain in the understanding of the cell composition, biological features, gene transcription regulation, and intercellular crosstalk within the intestinal system during disease states.

In the current study, we comprehensively profiled the landscape of cellular heterogeneity in HSCR patients with scRNA-seq. Subsequently, we validated our results using colon-derived organoids and stromal cells from human tissue, in addition to genetically modified experimental mouse models. This research unveiled a critical impairment in the regenerative crosstalk between the stromal and epithelial components, illuminating its significance in the development of HAEC. Our findings could have a substantial effect on the management and treatment of intestinal disorders in affected children, potentially enhancing outcomes and quality of life.

## Results

### Intestinal regeneration was impaired in the distal ganglionic colon of HSCR patients

**Fig. 1:**
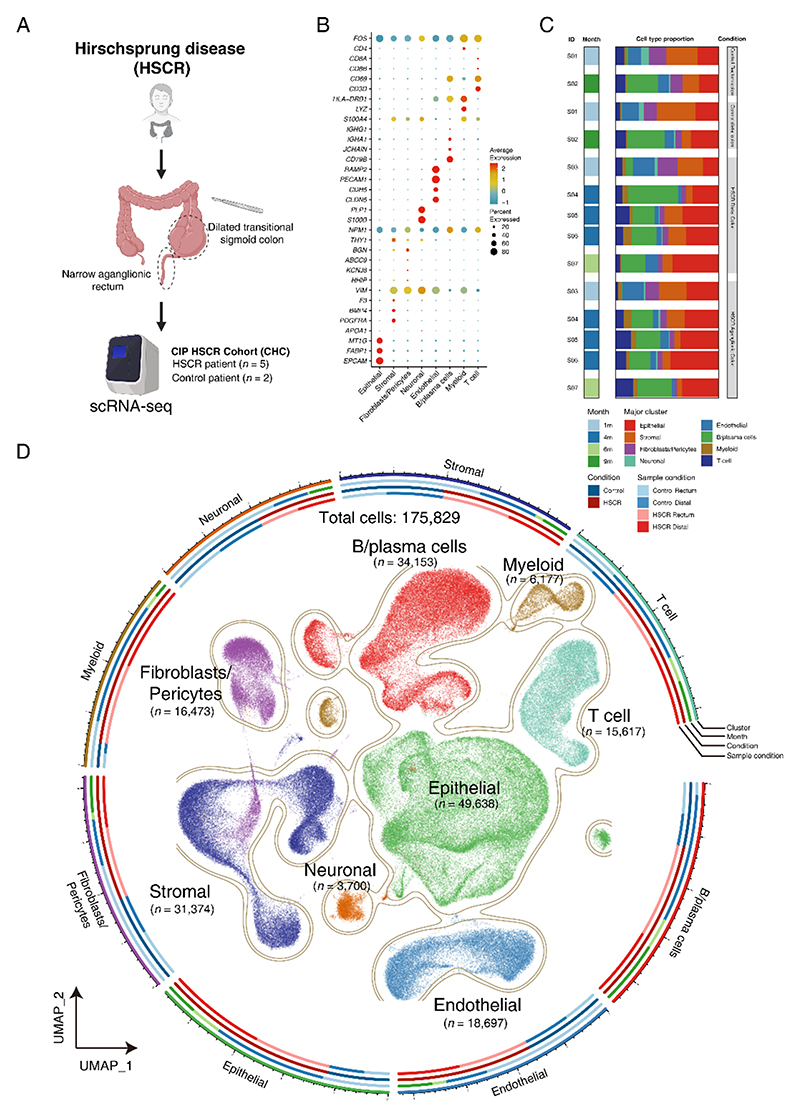
Study Design and analysis overview in Single-Cell RNA Sequencing **(A)** Overall study design with flowchart of single-cell analysis. **(B)** Markers of cell type specific genes used for cell annotation shown as fraction of expressing cells (circle size) and mean expression (color) of gene markers (rows) across major cell types (columns). **(C)** Illustrations showing the patients corresponding to the major cell types used for single cell RNA sequencing. **(D)** Uniform Manifold Approximation and Projection (UMAP) embedding of single cell transcriptomes of cells from eight major cell types.

To comprehensively explore the changes in cellular landscape in the colon of human HSCR patients, scRNA-seq of full-thickness colonic samples obtained from 5 HSCR patients and 2 non-HSCR control patients was performed (Fig. 1A), studying the aganglionic rectum (HSCR_R) and the distal ganglionic colon (HSCR_D) from HSCR patients, as well as the rectum (non-HSCR_R) and the distal colon (non-HSCR_D) from non-HSCR patients (Fig. 1B-C, S1A). After quality control, a total of 175,829 cells across all samples (HSCR_R (n = 60,461), HSCR_D (n = 56,930), non-HSCR_R (n = 27,558), and non-HSCR_D (n = 30,880)) were identified. Graph-based clustering and canonical cell marker annotation revealed eight major cell types, including epithelial cells (n = 49,638), fibroblasts/pericytes (n = 16,473), endothelial cells (n = 18,697), T cells (n = 15,617), B/plasma cells (n = 34,153), myeloid cells (n = 6,177), neuronal cells (n = 3,700), and stromal cells (n = 31,374) (Fig. 1D).

**Fig. 2:**
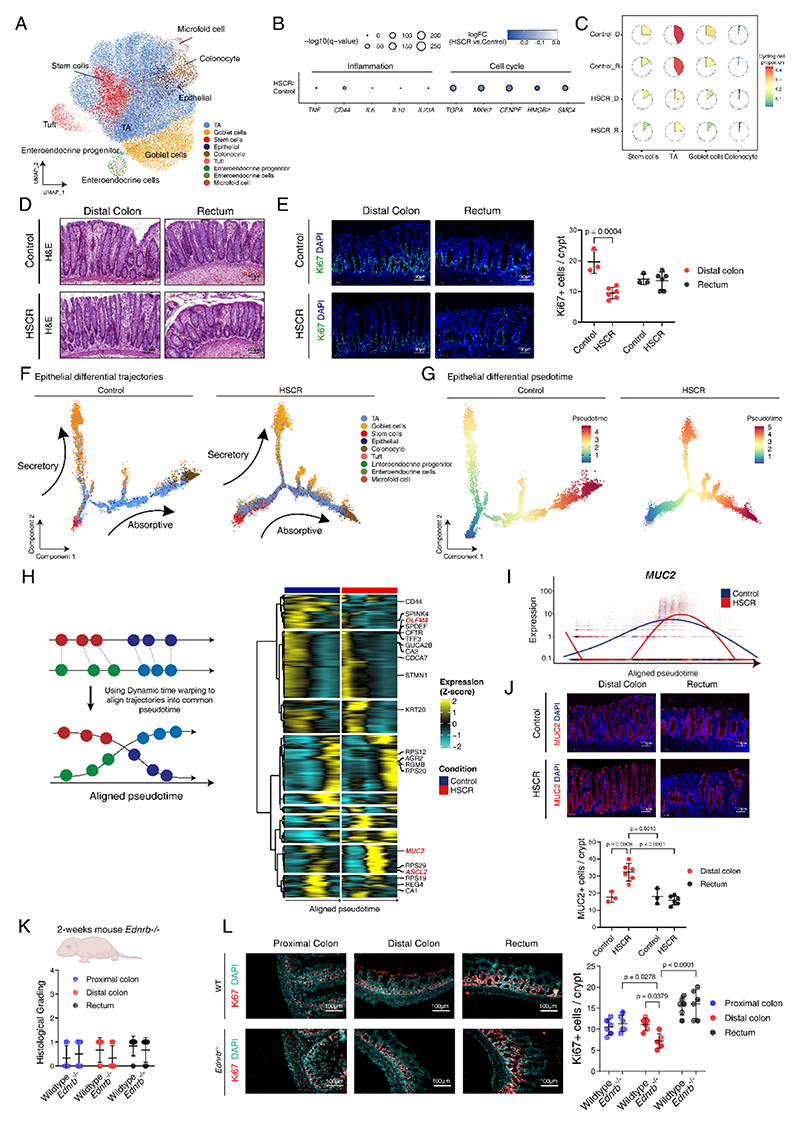
Impaired intestinal regeneration occurs in the colons of HSCR individual even in the absence of epithelial injury. **(A)** UMAP plot visualizing clusters of epithelial cell subtypes. **(B)** Dot plot of gene expression of markers in inflammation and cell cycle, with color indicating the fold change (log2 scale) comparing HSCR to control, and size indicating the adjusted p-value from Wilcoxon rank sum test. **(C)** Pie chart visualizing the proportion of cycling cells of different epithelial cell subtypes from different groups. **(D)** Representative H&E-stained histomicrographs of the distal colon and rectum from control/HSCR samples. **(E)** Immunofluorescence micrograph of Ki67 expression of the distal colon and rectum from control/HSCR samples, with DAPI nuclei counterstaining and quantification of Ki67 positive cells per crypt in all colonic regions. **(F)** Trajectory of epithelial cell subtypes in control and HSCR inferred by Monocle2. **(G)** Epithelial differential pseudotime in control and HSCR inferred by Monocle2. **(H)** Dynamic time warping of pseudotime trajectories of control and HSCR allows for comparison of the dynamics of epithelial differentiation process along a common axis. Hierarchical clustering of kinetic curves for dynamically regulated genes that vary significantly between control and HSCR epithelial trajectories. **(I)** *MUC2* gene expression across warped pseudotime in control and HSCR samples. Blue line as control and red line as HSCR. **(J)** Immunofluorescence micrograph of MUC2 expression of the distal colon and rectum from control/HSCR samples, with DAPI nuclei counterstaining and quantification. **(K)** Histological injury grading of the proximal colon, distal colon and rectum from 2-week-old wildtype and *Ednrb*^*-/-*^ mice. **(L)** Representative immunofluorescence staining for the proliferation marker Ki67 in the proximal colon, distal colon, and rectum from wildtype and *Ednrb*^*-/-*^ mice at 2 weeks, with DAPI nuclei counterstaining and quantification of Ki67+ cells per crypt. Scale bars are shown in all the images. HSCR patients n=6 and control patients n=3. Wildtype mice n=6 and *Ednrb*^*-/-*^ mice n=6. Experiments were repeated independently 3 times, with similar results. Each dots represented average value of each individual. Data are presented as mean ± SD and compared using one-way ANOVA with post-hoc tests. The primary p-value is indicated directly on the graph.

We first explored the compositional and transcriptional changes within the epithelial compartment. Of the 49,638 epithelial cells analyzed, various cell types were identified, including transit amplifying (TA), goblet cells, stem cells, epithelial, colonocyte, tuft, enteroendocrine progenitor, enteroendocrine cells, and microfold cells, based on marker genes (Fig. 2A, S1B). In the HSCR group, no changes were observed in inflammatory genes, while there was a significantly altered expression of cycling genes compared to controls (Fig. 2B). This was consistent with the absence of histological damage in the colonic epithelium but a reduced number of cycling cells across various epithelial cell types in HSCR, including stem cells, TA, and goblet cells (Fig. 2C-D). Additionally, the number of proliferating Ki67+ cells in HSCR patients within the crypts was reduced in comparison to the non-HSCR controls (Fig. 2E). To further understand the dynamic processes occurring within epithelial cells, we employed Monocle2 to perform trajectory analysis for both HSCR and controls. This trajectory illustrated differentiation pathways transitioning from stem cells/TAs either to goblet cells (the secretory branch) or to colonocytes (the absorptive branch) (Fig. 2F-G). To identify common transitions in the cell differentiation processes, despite variations in the timing of these processes between different cells or conditions, Dynamic Time Warping was used to align different trajectories onto a common pseudo-time axis (Fig. 2H, S1C-D). We found that mucin 2 (MUC2) expression peaked at a later stage in pseudo-time of the HSCR colon, signifying a delay in goblet cell differentiation and maturation (Fig. 2I). In accordance with the results from our scRNA-seq analysis, we observed an increased population of MUC2+ cells within the crypts of the HSCR distal colon (Fig. 2J). Building upon these observations in human samples, we sought to explore a similar phenomenon in mouse models, which provides the advantage of direct association of molecular alterations in HSCR that could eventually lead to HAEC-like epithelial damage. Utilizing *Ednrb*^*-/-*^ HSCR mice, there was an absence of the Hu-positive neuronal cells that characterize the aganglionic rectum (Fig. S2A-B). In line with previous research (40), no morphological damage and inflammation cytokine tumor necrosis factor alpha (*TNFα)* expression were found in the colon of 2-weeks old *Ednrb*^*-/-*^ mice (Fig. 2K, S2B-C). Similar to the HSCR patients, there was a reduction in proliferation in the distal colon of *Ednrb*^*-/-*^ mice compared to wildtype mice (Fig. 2L). These data suggest abnormal epithelial cell proliferation and differentiation in the distal ganglionic colon of individuals with HSCR, where no epithelial injury occurs.

**Fig. 3:**
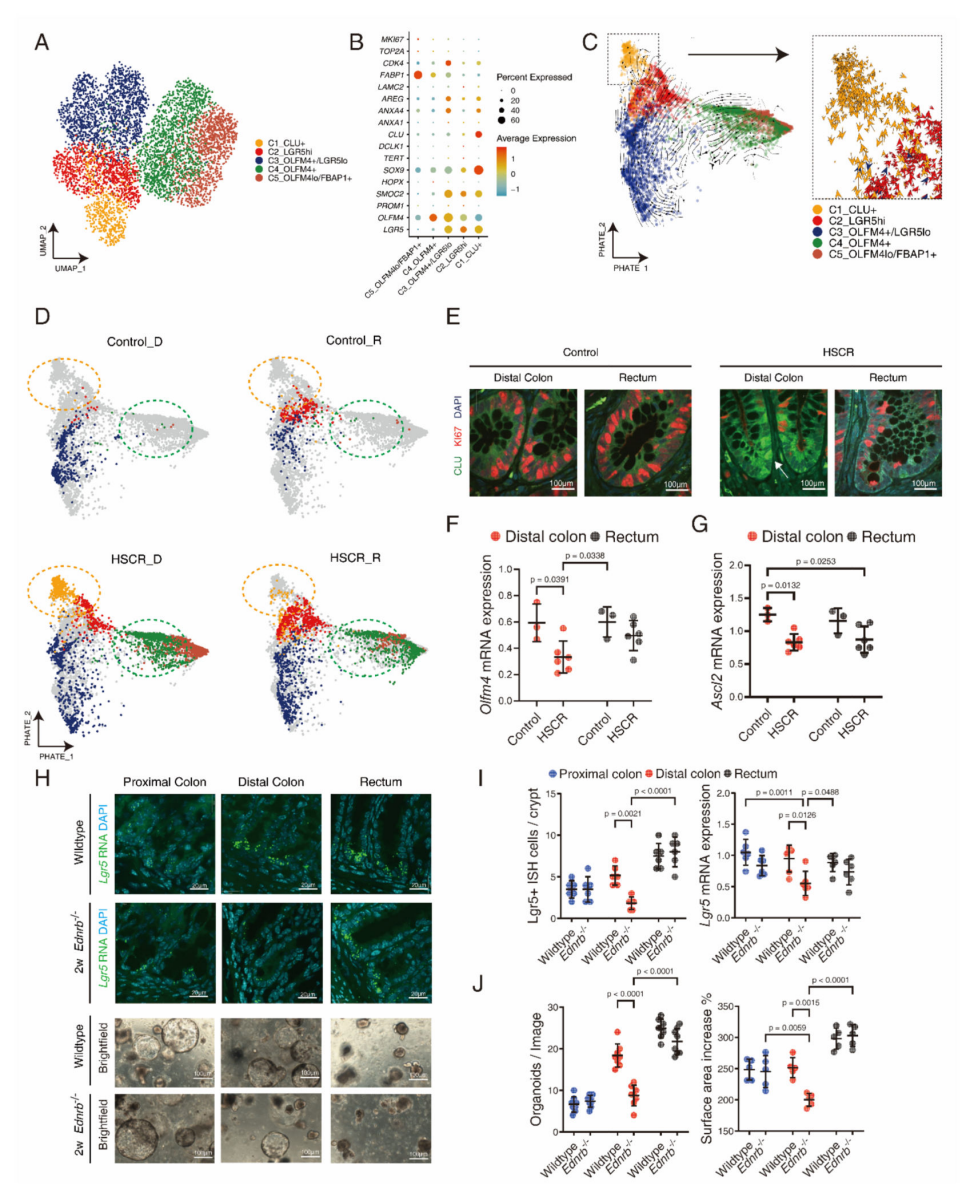
Alterations of intestinal stem cell dynamic occur in the distal colons of HSCR patients. **(A)** UMAP plot visualizing intestine stem cell populations. **(B)** Dot plot visualizing the average expression of ISC function-related markers. **(C)** PHATE plot visualizing intestine stem cell populations and overlaid with RNA velocity streams (arrows). **(D)** PHATE embedding of stem cell clusters in the distal and rectum of control and HSCR patients. **(E)** Representative micrographs of fluorescence staining for Clu (Green), Ki67 (Red) in the distal and rectum of control and HSCR patients, with DAPI nuclei counterstaining. **(F)** Quantification of *Olfm4* mRNA expression in the distal colon and rectum from control and HSCR samples. **(G)** Quantification of *ASCL2* mRNA expression in the distal colon and rectum from control and HSCR samples. **(H)** Representative fluorescence *in situ* hybridization (ISH) staining for *Lgr5* in the proximal colon, distal colon, and rectum from 2-week-old wildtype and *Ednrb*^*-/-*^ mice, with DAPI nuclei counterstaining and representative photomicrographs of organoids derived from proximal colon, distal colon and rectum of 2-week-old wildtype and *Ednrb*^*-/-*^ mice after 5 days in culture. **(I)** Quantification of Lgr5 positive ISH cells per crypt and Lgr5 mRNA expression in the proximal colon, distal colon and rectum from 2-week-old wildtype and *Ednrb*^*-/-*^ mice. **(J)** Quantification of organoids number in each image and percentage of increase in surface area from day 2 to day 5 cultures. Quantification of surface area increase comparing day 5 to day 2 of all groups from 2-week-old wildtype and *Ednrb*^*-/-*^ mice. Scale bars are shown in all the images. HSCR patients n=6 and control patients n=3. Wildtype mice n=6 and *Ednrb*^*-/-*^ mice n=6. Experiments were repeated independently 3 times, with similar results. Each dots represented average value of each individual. Data are presented as mean ± SD and compared using one-way ANOVA with post-hoc tests. The primary p-value is indicated directly on the graph.

To investigate the heterogeneity of intestinal stem cells (ISCs) in the HSCR patients, we conducted a focused analysis of the stem cell niche, including a total of 5437 stem cells. These were categorized into five clusters based on the expression levels of key stemness markers (CLU, LGR5, OLFM4) (Fig. 3A-B). Employing the PHATE method, a more effective visualization of the developmental trajectories of the stem cell clusters was achieved. The examination revealed the presence of two distinct sources for stem cell clusters, as evidenced by RNA velocity streams. The initial source of ISC involves the LGR5+ and OLFM4+/LGR5low population (Fig. 3C-D, S3A-C), characterized by substantial expansion leading to the emergence of the OLFM4+ population, and OLFM4+/FABP1. This pattern correlates with LGR5 being a well-recognized marker of active colonic stem cell progenitors (7), and OLFM4 is broadly expressed by crypt cells including LGR5-positive stem cells and the transit-amplifying progenitor cells located above LGR5-positive cells (41). Interestingly, the CLU+ population emerges as the second source of stem cells, giving rise to LGR5+ ISC (Fig. 3C-D, S3A-C), which aligns with recent discoveries of CLU+ ‘revival stem cells’ that can be triggered in response to tissue damage to repopulate all epithelial cell types (13). Most strikingly, the present findings demonstrated a predominant presence of CLU+ stem cell clusters in the HSCR distal colon segment (Fig. 3D-E, S3C), even though they are reported rarely found in a healthy intestine (13). Furthermore, compared to controls, the expression of intestinal stem cell markers achaete-scute complex homolog 2 (*ASCL2*) and olfactomedin 4 (*OLFM4*) in the HSCR colon declined at an earlier pseudo-time, indicating a compromised stem cell niche (Fig. 3F-G). Moreover, the expression levels of +4 reserve stem cell (RSC) markers (*BMI1, HOPX*, and *TERT*) (10–12) were notably low, suggesting that they may not play a substantial role in this circumstance (Fig. S4A, B). In addition, in the distal ganglionic colon of *Ednrb*^*-/-*^ 2-week-old mice, *Lgr5* ISC expression was decreased compared to the wildtype mice (Fig. 3H-I). The organoids derived from distal ganglionic colon demonstrated a reduction in number and size compared to the organoids derived from wildtype distal colon, indicating an overall decrease in epithelial organoid growth and regeneration. (Fig. 3H, J). Collectively, these results showcase the impairment of intestinal regeneration and distinct dynamic ISCs in the distal colon of individuals with HSCR, where no observable injury was detected in the epithelium.

### Impaired intestinal regeneration in the distal ganglionic colon of HAEC

**Fig. 4:**
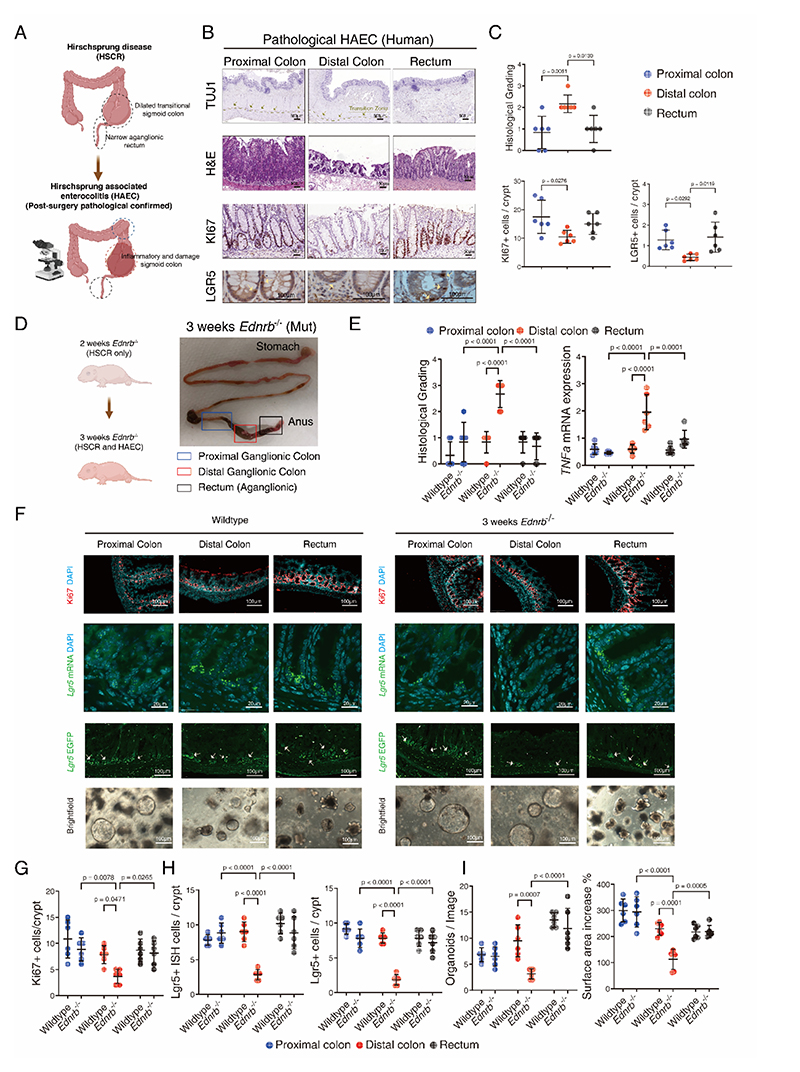
Impairment of intestinal regeneration in the colon of HAEC patients and mice with HAEC. **(A)** Schematic representation of HAEC pathology/diagnosis, as determined by histological assessment. **(B)** Representative neuron marker TUJ1 stained, H&E-stained, Ki67-stained and Lgr5-stained histomicrographs in the proximal, distal and aganglionic rectum from HAEC patients. **(C)** Histological injury grading scores, quantification of Ki67 positive cells per crypt and quantification of Lgr5 positive cells per crypt of the proximal, distal and rectum from HAEC patients. **(D)** Representative images of mouse morphology and dissected whole gastrointestinal tract of *Ednrb*^*-/-*^ mouse at 3-weeks of age. Black box: proximal colon, red box: dilated distal ganglionic colon, and green box: rectum; and their corresponding segments in wildtype colon. **(E)** Histological injury grading scores and pro-inflammation maker *TNFa* mRNA expression of the proximal, distal and rectum of *Ednrb*^*-/-*^ mouse at 3-weeks of age. **(F)** Immunofluorescence staining for the proliferation marker Ki67, *in situ* hybridization (ISH) staining for *Lgr5*, intestinal stem cell marker Lgr5 in the proximal colon, distal colon and rectum from wildtype and *Ednrb*^*-/-*^ mice at 3-week-old, with DAPI nuclei counterstaining, and representative photomicrographs of organoids derived from proximal colon, distal colon and rectum of wildtype and *Ednrb*^*-/-*^ mice after 5 days in culture. **(G)** Quantification of Ki67+ cells per crypt in the proximal colon, distal colon and rectum from wildtype and *Ednrb*^*-/-*^ mice. **(H)** Quantification of *Lgr5* ISC positive cells per crypt and quantification of Lgr5-GFP+ cells per crypt in the proximal colon, distal colon and rectum. **(I)** Quantification of organoids number in each image and percentage of increase in surface area from day 2 to day 5 cultures of all groups from wildtype and *Ednrb*^*-/-*^ mice. Scale bars are shown in all the images. HSCR patients n=6 and control patients n=3. Wildtype mice n=6 and *Ednrb*^*-/-*^ mice n=6. Experiments were repeated independently 3 times, with similar results. Each dots represented average value of each individual. Data are presented as mean ± SD and compared using one-way ANOVA with post-hoc tests. The primary p-value is indicated directly on the graph.

To further validate these experimental observations and evaluate the intestinal regeneration ability in HSCR patients proceeding to HAEC. Colonic samples from six patients diagnosed with HAEC (based on histological assessments conducted by pathologists) were analyzed (Fig. 4A-B). There was an observed damage to the distal ganglionic colonic epithelium when compared to both the proximal ganglionic colon and aganglionic rectum (Fig. 4B-C). Within the same distal colonic segment, there was diminished expression of both proliferation marker Ki67 and the intestinal stem cell marker LGR5 (Fig. 4B-C, S5A-B).

Aganglionosis of *Ednrb*^*-/-*^ HSCR mice led to the eventual colonic dilatation observed at 3-week-old. The distal ganglionic colon in *Ednrb*^*-/-*^ mice exhibited severe epithelial injury, along with an upregulation of the inflammatory marker *TNFα* gene (Fig. 4D-E, S5C-D). The distal ganglionic colon of *Ednrb*^*-/-*^ mice showed a significant decrease in Ki67+ proliferative cells (Fig. 4F-G), as well as decrease in LGR5 active intestinal stem cells (Fig. 4F, H, S5E). Additionally, organoids cultured from the distal ganglionic colon of *Ednrb*^*-/-*^ mice were characterized by both a reduction in number and size in comparison to organoids derived from the distal colon of wildtype mice (Fig. 4F, I), thus demonstrating defective regeneration in the distal ganglionic colon of *Ednrb*^*-/-*^ mice at 3 weeks. Taken together, these findings confirm the impairment of intestinal regeneration in the distal ganglionic colon affected by HAEC, which progresses from HSCR individuals.

### The colons of HSCR patients experience a shift in stromal cell composition

**Fig. 5:**
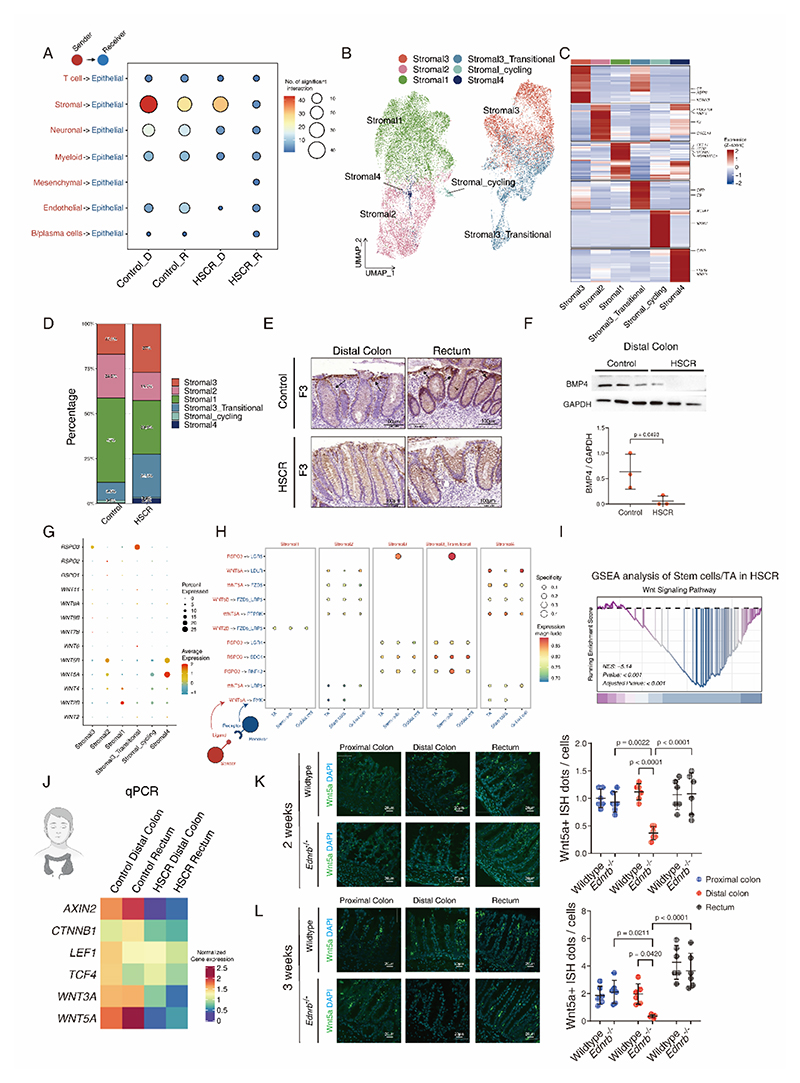
Wnt-releasing Stromal 2 cell population was reduced in the colon of HSCR patients. **(A)** Dot plot visualizing the interaction strength between ligands expressed by other cell types with receptors expressed by epithelial cells. **(B)** UMAP plot visualizing clusters of stromal cell subtypes. **(C)** Heatmap visualizing gene expression of stromal cell subtype specific markers. **(D)** Proportion of stromal cell subtypes in control and HSCR samples. **(E)** Representative histomicrographs of Stromal 2 cell marker F3 of the distal colon and rectum from control and HSCR samples. **(F)** Protein expression of Stromal 2 cell marker BMP4 of control and HSCR samples (n=3). **(G)** Dot plot of WNT signaling gene expression in different stromal cell subtypes. **(H)** Dot plot of ligand-receptor pairs in WNT signaling between stromal cells and epithelial cells. **(I)** GSEA analysis shows WNT signalling is impaired in stem cells/TAs in HSCR group. **(J)** Quantification of tissue expression of Wnt pathway genes in the distal and rectum from control and HSCR. **(K)** Representative micrographs of fluorescence ISH staining for *Wnt5a* in the proximal, distal, and rectum from 2 weeks old wildtype and *Ednrb*^*-/-*^ mice, with DAPI nuclei counterstaining and quantification of ISH of Wnt5a positive dots per cells. **(L)** Representative micrographs of fluorescence ISH staining for *Wnt5a* and quantification of ISH of *Wnt5a* positive dots per cell in the proximal colon, distal colon and rectum from 3 weeks old wildtype and *Ednrb*^*-/-*^ mice. Scale bars are shown in all the images. HSCR patients n=6 and control patients n=3. Wildtype mice n=6 and *Ednrb*^*-/-*^ mice n=6. Experiments were repeated independently 3 times, with similar results. Each dots represented average value of each individual. Data are presented as mean ± SD and compared using nonparametric unpaired Student’s t-tests or one-way ANOVA with post-hoc tests as appropriate. The primary p-value is indicated directly on the graph.

The viability and regenerative capacity of the intestinal epithelium depend on the coordinated interactions between the diverse cellular components within the intestinal tissue. To examine these interactions and to identify the specific cell population responsible for the changes observed in the HSCR colonic epithelium, a detailed analysis of cell-to-cell communication was conducted. By studying the interactions between receptors expressed on epithelial cells with ligands secreted by other intestinal cell types, we found the strongest interaction exists from stromal cells to epithelial cells (Fig. 5A, S6A), suggesting a significant regulatory role of stromal cells on the colonic epithelium through ligand secretion. Building upon this insight, subsequent investigations through unsupervised clustering unveiled the presence of five distinct sub-types of stromal cells (Fig. 5B-C), characterized using previously reported specific markers (19). This analysis revealed a notable reduction in both Stromal 1 and Stromal 2 cell population in HSCR samples compare to non-HSCR samples (Fig. 5D), with Stromal 2 cells characterized by the presence of markers including platelet-derived growth factor receptor alpha (*PDGFRα*), coagulation factor 3 (*F3*), and bone morphogenetic protein 4 (*BMP4*). To further validate these findings, we performed additional F3 and BMP4 protein analysis, which confirmed a significant decrease in the expression levels of these critical Stromal 2 cell markers among HSCR patients (Fig. 5E-F).

Stromal 2 cells are well-known for their role as a primary source of WNT signaling in maintaining the epithelial stem cell niche and regenerative ability. Therefore, to evaluate the correlation between the diminished Stromal 2 cell population and WNT activity, we conducted a stromal cell subtype specific expression analysis of genes related to the Wnt/β-catenin pathway (Fig. 5G), revealing Stromal 2 as a Wnt expressing stromal cell. Through a comprehensive examination of cell-to-cell interactions, we identified specific ligand-receptor interactions within the Wnt/β-catenin pathway (Fig. 5H). Strikingly, Stromal 2 cells secrete WNT5A ligands, which binds to epithelial receptors including frizzled class receptor 5 (FZD5), low-density lipoprotein receptor-related protein 5 (LRP5) and receptor like tyrosine kinase (RYK), facilitating communication between stromal and epithelial cells. Additionally, Gene set enrichment analysis (GSEA) revealed a significant reduction in Wnt/β-catenin pathway activity in the HSCR colon (Fig. 5I). Whole tissue gene expression analysis provided additional confirmation of the decreased activity in the Wnt/β-catenin pathway in HSCR patients (Fig. 5J), thus we extended further to ascertain if the Wnt pathway activity was compromised in the HSCR mouse model. Colonic crypt expression level of *Wnt5a* was reduced in the distal ganglionic colon of both 2-week-old and 3-week-old *Ednrb*^*-/-*^ mice (Fig. 5K-L). This collective evidence underscores a scene that Wnt signaling decrease in HSCR was attributed to the reduction in the Stromal 2 cell population, potentially leading to the epithelial regenerative impairment.

**Fig. 6:**
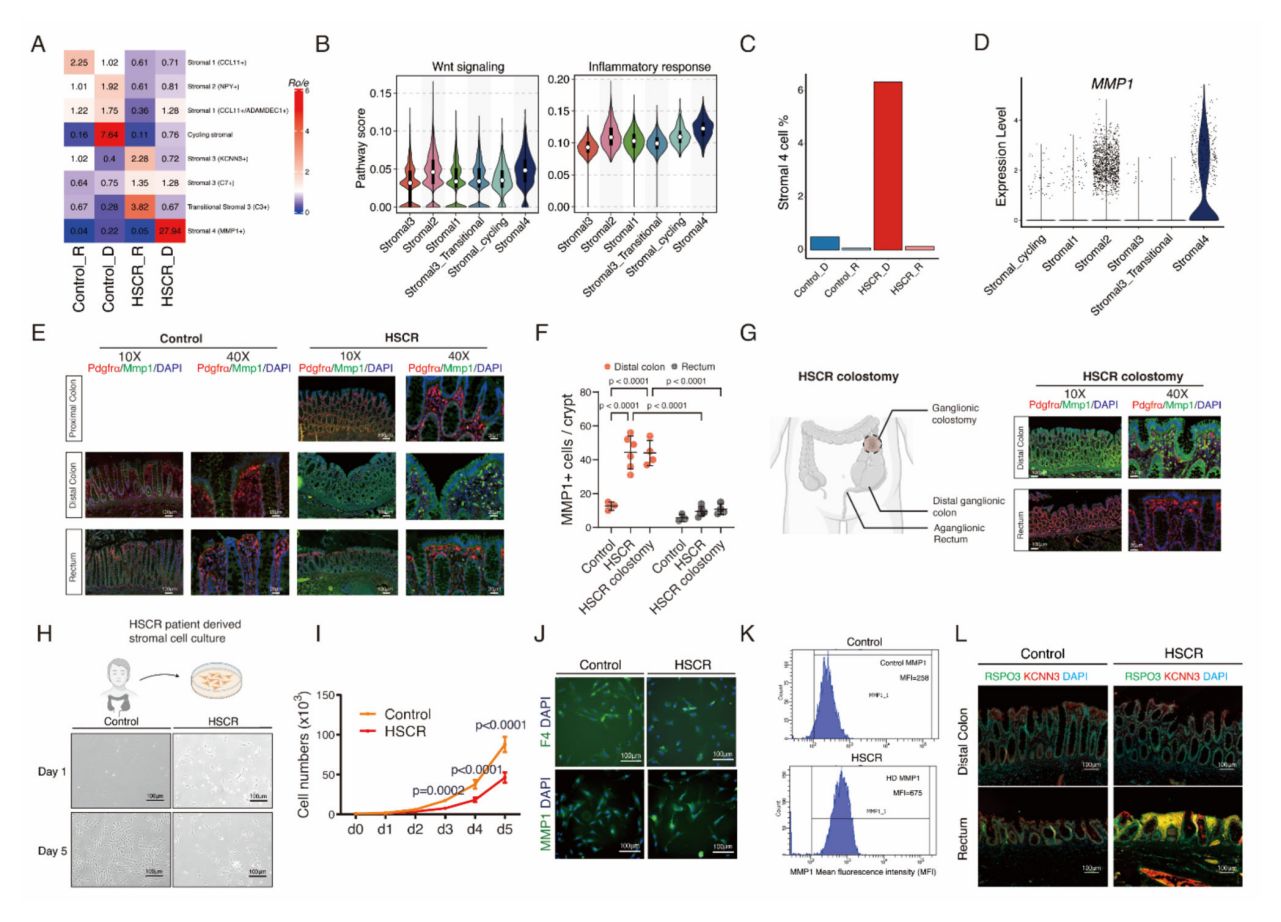
Pro-inflammatory Stromal 4 cells were highly expressed in the distal colon of HSCR patients **(A)** Heatmap visualizing the enrichment of specific stromal cell subtype among the different groups studied, estimated by R_o/e_. **(B)** Violin plots showing activity of signaling pathway inferred by UCell in different stromal cell subtypes. **(C)** Bar chart showing the percentage of Stromal 4 cells to total number of stromal cells in different groups. **(D)** Violin plots showing MMP1 expression in different stromal cell subtypes. **(E)** Representative immuno-staining micrographs of Pdgfrα (red) and MMP1 (green) in the colon from non-HSCR control and HSCR patients, with DAPI nuclei counterstaining and **(F)** quantification of MMP1 positive cells per crypt of control and HSCR patients. **(G)** Representative immuno-staining micrographs of Pdgfrα (red) and MMP1 (green) in the biopsy of obtained from the distal ganglionic colon and aganglionic rectum during pull-through surgery from HSCR patients following colostomy (DAPI nuclei counterstaining). **(H)** Representative photomicrographs of stromal cells derived from distal colon from non-HSCR control and HSCR patients at day 1 and day 5 of culture. **(I)** Growth curve of stromal cells derived from distal colon of non-HSCR control and HSCR patients, measured by cell number quantified daily for 5 days. **(J)** Immunofluorescence micrograph of F4+ Stromal 2 cells and MMP1+ Stromal 4 cells in the stromal cell culture derived from distal colon of non-HSCR and HSCR patients, with DAPI nuclei counterstaining. **(K)** Mean fluorescence intensity (MFI) of MMP1 in the stromal cell culture derived from distal colon of non-HSCR and HSCR patients. **(L)** Representative immunofluorescence micrographs of stromal cells 3 marker RSPO3 (Green) and KCNN3 (Red) in the distal and rectum from HSCR patient and non-HSCR control patient, with DAPI nuclei counterstaining. Scale bars are shown in all the images. HSCR patients n=6 and control patients n=3. Experiments were repeated independently 3 times, with similar results. Each dots represented average value of each individual. Data are presented as mean ± SD and compared using nonparametric unpaired Student’s t-tests or one-way ANOVA with post-hoc tests as appropriate. The primary p-value is indicated directly on the graph.

Further quantification of the observed-to-expected cell number ratios highlighted the substantial presence of Stromal 4 cells specifically in the distal colon of HSCR patients, underscoring their crucial role in this region (Fig. 6A). Stromal 4 cells have previously been linked to inflammation and are typically observed only in inflamed intestinal tissue (19, 42). Surprisingly, the Stromal 4 cells exhibited a pro-inflammatory state as demonstrated by pathway scoring of stromal subtypes (Fig. 6B), contrasting with the absence of epithelial inflammation in the same segment at this stage of HSCR (Fig. 2B). Our investigation revealed the presence of Stromal 4 cells exclusively in the distal ganglionic colon of HSCR patients, supported by the highest expression of the matrix metallopeptidase 1 (MMP1) marker in the same segment (Fig. 6C), but depletion of the stromal cell 2 marker Pdgfrα (Fig. 6E, F). In addition, we confirmed that MMP1 are absent in the proximal ganglionic colon at the level of the defunctioning colostomy but conversely present in the stromal cells from the distal ganglionic colon (Fig. 6F, G). Subsequently, we isolated and cultured the stromal cells derived from the distal colon of both HSCR patients and non-HSCR controls. Notably, there was a markedly reduced stromal cell growth in HSCR patients as compared to non-HSCR controls (Fig. 6H, I). Furthermore, there was an observed decrease in the number of Stromal 2 marker F4-positive cells (Fig. 6J) and an increase in the number of Stromal 4 marker MMP1-positive cells in the stromal cell culture derived from HSCR patients (Fig. 6J, K). In contrast, Stromal 4 cells were exclusively expressed in the distal colon, while Stromal 3 cells were limited to the aganglionic rectum in HSCR patients (Fig. 6L, S6B). Overall, the colons of HSCR patients exhibit a significant shift in stromal cell composition, characterized by a reduction in Stromal 2 cells and an exclusive increase in Stromal 4 cells in the distal colon, along with an exclusive presence of Stromal 3 cells in the rectum.

### The failure of Stromal cells from the distal colon of HSCR patients to support the growth of epithelial organoids can be rescued by inhibiting Stromal 4 cells

**Fig. 7:**
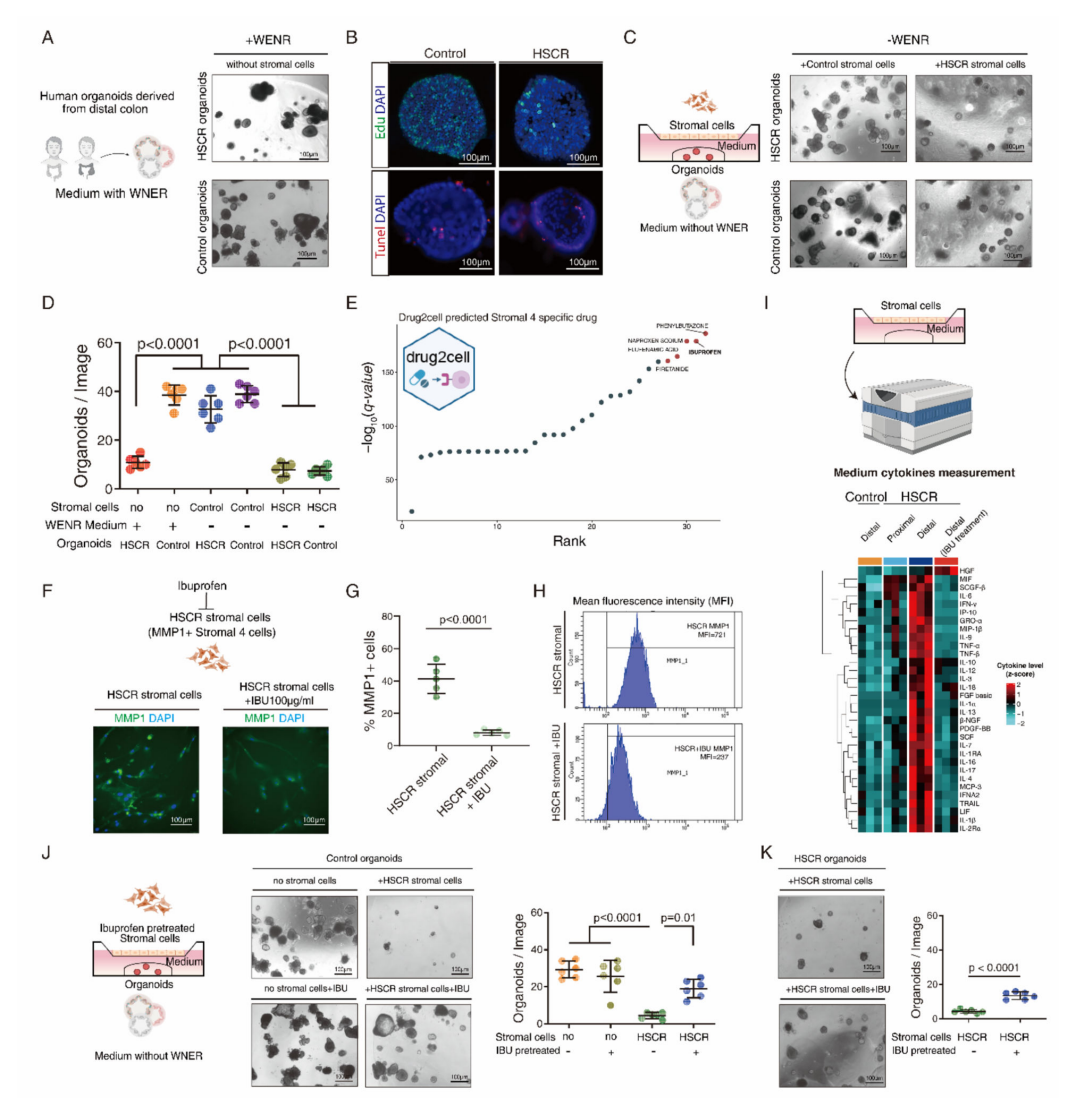
The failure of Stromal cells from the distal colon of HSCR patients to support the growth of epithelial organoids can be rescued by inhibiting Stromal 4 cells **(A)** Representative photomicrographs of distal colon epithelial organoids derived from non-HSCR control and HSCR patients, cultured in media enriched with recombinant “WENR” growth factors Wnt3a, epidermal growth factor (EGF), Noggin, and Rspo1. **(B)** Representative photomicrographs of EDU and TUNEL staining for distal colon epithelial organoids derived from non-HSCR control and HSCR patients cultured with WENR growth factors. **(C)** Representative photomicrographs of distal colon epithelial organoids derived from non-HSCR control and HSCR patients, co-cultured with the distal colon stromal cell derived from non-HSCR control and HSCR patients in the absence of WENR growth factors. **(D)** Quantification of organoids number/ image in each culturing conditions. **(E)** Drug2cell analysis prediction of specific drugs inhibiting Stromal 4 cells (Wilcoxon rank sum test, q-value: FDR-adjusted p-value). **(F)** Immunofluorescence micrograph of MMP1+ Stromal 4 cells derived from HSCR patient’s distal colon treated with or without ibuprofen (Ibu, 100µg/mL), with DAPI nuclei counterstaining. **(G)** Quantification percentage of MMP1 positive cells over total number of HSCR distal colon stromal cells treated with or without ibuprofen. **(H)** Mean fluorescence intensity (MFI) of MMP1 in the HSCR distal colon stromal cell treated with or without ibuprofen. **(I)** Cytokines released from stromal cells from HSCR proximal colon, distal colon treated with or without ibuprofen as well as the non-HSCR control distal colon. **(J)** Representative photomicrographs of non-HSCR control epithelial organoids co-cultured with HSCR distal colon stromal cells pretreated with or without ibuprofen in the absence of WENR growth factors and quantification of organoids number in each culturing condition. **(K)** Representative photomicrographs of HSCR organoids co-cultured with HSCR distal colon stromal cells pretreated with or without ibuprofen in the absence of WENR growth factors and quantification of organoids number in each culturing condition. Scale bars are shown in all the images. HSCR patients n=6 and control patients n=3. Experiments were repeated independently 3 times, with similar results. Each dots represented average value of each individual. Data are presented as mean ± SD and compared using nonparametric unpaired Student’s t-tests or one-way ANOVA with post-hoc tests as appropriate. The primary p-value is indicated directly on the graph.

To verify the contribution of Stromal 4 cells to colonic epithelial regenerative defects, we established an *in vitro* cell-organoids co-culture model to study the interaction between stromal cells and colonic organoids. Epithelial organoids generated from the distal ganglionic colonic epithelium of HSCR patients exhibited impaired growth compared to those derived from control patients (Fig. 7A, S7A), similar to what was observed in organoids derived from HSCR disease mouse model (Fig. 3H). This growth impairment in organoids, characterized by reduced proliferation resembling the tissue phenotype but not caused by apoptosis (Fig. 7B), does not compromise their ability to self-assemble into complex epithelial assembloids (Fig. S7). Remarkably, when these organoids were co-cultured with stromal cells using a transwell system, the stromal cells from control patients effectively supported the growth of the distal ganglionic colonic organoids from HSCR patients (Fig. 7C, D, S7B). In contrast, the HSCR stromal cells were unable to provide sufficient support for control patient-derived organoid growth. These findings demonstrate that the expression of pro-inflammatory Stromal 4 cells in the distal colon of HSCR patients is insufficient to facilitate the growth of epithelial cells and impedes the regenerative ability of the colonic epithelium, which may contribute to the development HAEC epithelial injury.

In order to discover potential strategies for preventing the impairment of epithelial regeneration due to Stromal 4 cells, we conducted drug-to-cell predictions aimed at suppressing the Stromal 4 cell population specifically. Ibuprofen was one of the top candidates based on bioactivity of drugs available in the ChEMBL database and their specificity in targeting Stromal 4 cells compared to other cell types of the HSCR colon (Fig. 7E). Administration of ibuprofen to stromal cells isolated from the distal colon of HSCR patients resulted in an effective reduction of MMP1-positive Stromal 4 cells (Fig. 7F, G), along with a reduction in the intracellular intensity of MMP1 within the stromal cells (Fig. 7H). Cytokine profiles secreted by stromal cells was found significantly higher levels of proinflammatory cytokines from HSCR distal ganglionic colon cells compared to proximal ganglionic and non-HSCR controls. Excitingly, pretreatment of these stromal cells with Ibuprofen reversed the cytokine profile to control levels (Fig. 7I). HSCR distal colon stromal cells exhibited an inability to facilitate the growth of organoids. Conversely, HSCR distal colon stromal cells that were pre-treated with ibuprofen were capable of supporting the growth of control colonic epithelial organoids (Fig. 7J, K), suggesting a positive impact of ibuprofen on the ability of HSCR stromal cells to support organoid growth. Notably, ibuprofen treatment directly on organoids did not show any effects when stromal cells were lacking (Fig. 7J, K). This implies that the positive effects of ibuprofen are associated with its interaction with stromal cells rather than a direct impact on the organoids. These findings suggest that ibuprofen holds promise as a potential therapeutic candidate for preventing the progression to HAEC through mitigating the disruption of epithelial regeneration in HSCR by inhibiting pro-inflammatory stromal cell population.

## Discussion

HSCR is a devastating life-threatening disorder often associated with HAEC, which remains poorly understood. This study provides four key insights: (1) Despite the absence of epithelial damage, both HSCR patients and experimental HSCR mice consistently exhibited impaired epithelial regeneration in the distal ganglionic colon. (2) This impaired regeneration ability precedes HAEC when epithelial damage occurs on-site. (3) HSCR patients show a shift in the type of stromal cells, with fewer Wnt-releasing Stromal 2 cells and the exclusive presence of pro-inflammatory Stromal 4 cells in the distal ganglionic colon. (4) Stromal cells derived from the distal ganglionic colon of HSCR patients were unable to sustain healthy organoid growth. Remarkably, the application of ibuprofen inhibited Stromal 4 cells and successfully restored epithelial organoid growth.

In this study, we observed a significant decrease in the active stem cell population and epithelial proliferation in the distal ganglionic colon of both experimental HSCR mice and HSCR mice progressing to HAEC. These findings align with previous research demonstrating reduced epithelial cell proliferation in the ganglionic segments of *Ednrb* mutant mice compared to wildtype mice (32). Moreover, we found a similar phenotype in resected colonic tissue from HSCR pull through operation, as well as in resected colonic tissue from HSCR cases where the presence of epithelial damage was confirmed by post-operative pathological assessment. Such observations regarding the reduction of Ki67-positive proliferating cells in the intestine are more commonly associated with neonatal intestinal injuries like necrotizing enterocolitis (43–45), contrasting with the hyperproliferative state typically seen in colonic tissue affected by inflammatory bowel disease (43–45). Early diagnosis and prevention of HAEC pose significant challenges due to its complexity. It is plausible that the seemingly healthy segment of the ganglionic colon, where no apparent epithelial damage is evident, may exhibit significantly reduced levels of Wnt/β-catenin signaling and diminished regenerative capacity. This reduction in regenerative capacity could increase the risk of HAEC development by limiting the ability of the colonic epithelium to respond effectively to insults. As previously observed (40), the morphological changes induced by HAEC and the impairment of intestinal regeneration during the early stages (*Ednrb*^*-/-*^ mice at 2 weeks of age) are subtle. This research demonstrates that organoids derived from the colonic epithelium exhibit exceptional sensitivity in detecting early epithelial alterations and regenerative impairment. Therefore, organoids obtained from HSCR patients’ surgically removed colon tissue could potentially be used as a post-operative screening tool to assess the risk of developing HAEC. Another important finding pertains to the phenotype of goblet cells. Traditionally, goblet cells were considered non-cycling, fully differentiated cells. However, recent reports show that MUC2-positive cells also express the proliferation-associated marker HMGB2 within the Transit-amplifying (TA) zones of healthy adult colonic tissues, suggesting that cell type specification can occur even in proliferating TA cells (46). Interestingly, the proliferative state of these cycling goblet cells was altered in ulcerative colitis (46). Our current study echoes these findings, showing that despite the altered expression of MUC2 mRNA and protein in HSCR tissues, the proportion of cycling goblet cells was lower than in control, indicating a shift in goblet cell dynamics. These results deepen our understanding of the diseases, as mucin production is essential for maintaining intestinal barrier function. Investigating the mechanisms and role of goblet cells in the pathogenesis of HSCR and associated enterocolitis is a focus we intend to pursue in future directions of our study.

Recent studies have highlighted the intestinal regenerative program driven by the Clu revival stem cell, facilitating fetal reprogramming in response to injuries such as irradiation or DSS-induced colitis (13), as well as in cancer studies (47, 48). These findings suggest a predominant presence of CLU+ stem cell clusters in HSCR tissues, particularly in the HSCR distal colon segment. This CLU+ population demonstrated the capacity to give rise to LGR5+ cells, supporting the hypothesis that CLU+ stem cells possess the capacity to generate diverse stem cell populations (13). Strikingly, the current state of the epithelium in this region appears relatively normal, showing no detectable injury but posing a higher risk of injury development in the future. The regenerative reprogramming of ISCs occurs prior to the onset of injury, concurrently with stromal preconditioning, showcasing a remarkable plasticity capability in the neonatal intestine. Notably, the +4 reserve stem cells (RSC) do not appear to contribute significantly to this reprogramming process. A remarkable property of regenerating intestinal epithelium is its acquisition of a fetal-like regenerative state and subsequent regenerative process that has been reported (48, 49). While Clu and Stromal 4 have both been observed in fetal gut (37, 50), we found a notably low expression of fetal distal colon progenitors (50), suggesting that the dynamic changes in ISCs in the distal colon of HSCR are not attributed to developmental delays but are instead highly reliant on microenvironmental changes that facilitate this fetal-like reprogramming. Future preventive measures for intestinal injury in the distal colon of HSCR may involve understanding and manipulating the intestinal crypt microenvironment to support the fetal-like reprogramming regenerative capabilities observed in the neonatal intestine.

The Wnt signaling pathway has been recognized for its crucial role in perinatal and postnatal epithelial development (51, 52). In this study, we further elucidated the etiology surrounding the aberrant Wnt expression in the HSCR dilated distal ganglionic colon. Within the complex landscape of the intestinal microenvironment, stromal cells emerge as key players in supporting epithelial stem cell function and tissue homeostasis (53). Among the stromal cell subtypes, Stromal 2 cells, characterized by their expression of F3/CD142 and crucial Wnt genes, have garnered significant attention (19, 54, 55). These cells, located in close proximity to the epithelial crypts, demonstrate a high expression of transforming growth factor β (TGF-β) superfamily ligands such as BMP2 and BMP5, non-canonical Wnt ligands including WNT5A and WNT5B, as well as the secreted Wnt antagonist frizzled related protein (FRZB) (19). Notably, WNT5A has been identified as an essential factor for epithelial reconstitution after injury (56). The combined secretion of these factors by Stromal 2 cells suggests their critical role in supporting epithelial stem cell proliferation, differentiation, and maintaining the mesenchymal niche. Additionally, PDGFRα+ stromal cells are well-established for their pivotal role in the intestinal stroma in experimental mice study (57, 58). These cells serve as the primary source of essential factors, including WNTs and R-spondin 3 (RSPO3), critical for the maintenance of murine intestinal stem cells *in vivo* (57). Furthermore, PDGFRα-induced stromal maturation is crucial for regulating postnatal intestinal epithelial stemness and enhancing defense mechanisms (58). The cell annotation applied in this study distinctly characterized PDGFRα+ cells as Stromal 2 cells in the current study, further underscoring that PDGFRα-expressing stromal cells and Stromal 2 cells are indeed the same population, but divergent between human and mice. Our findings are consistent with previous research (19), demonstrating a significant reduction in F3+ Stromal 2 cells in individuals with HSCR compared to control samples. A consistent decrease in Wnt5a levels in both patients with HSCR and the HSCR mouse model was also observed. Most strikingly, human HSCR distal colon-derived stromal cells showed reduced Stromal 2 population, which not only failed to support the growth of healthy organoids but also exhibited a marked impairment in maintaining the epithelial stem cell niche and regenerative capacity (19). These findings underscore the critical role of Stromal 2 cells and Wnt5a in maintaining intestinal health, and further highlight their potential as therapeutic targets in HSCR. It is essential to consider the involvement of other factors within the complex intestinal microenvironment. Macrophages, particularly anti-inflammatory M2 macrophages, serve a critical function in releasing Wnt in response to epithelial injury (21, 22); however, in the dilated colon of HAEC patients and mice with HAEC like injuries, a notable failure to transition pro-inflammatory M1 to anti-inflammatory M2 macrophages has been observed (40). This impaired maturation of macrophages may potentially contribute to the decrease in Wnt expression (59), suggesting a possible role for macrophage dysfunction in releasing Wnt in the HSCR distal colon. Interestingly, HAEC disappears with age clinically, which is very likely due to the maturation of Wnt/stem cells activity in the intestine. These intriguing findings underscore the complexity of the intestinal ecosystem and prompt further investigations to elucidate the interplay among various cellular components, signaling pathways, and environmental factors that collectively influence intestinal health and disease.

Changes in the ulcerative colitis stroma encompass a reduction in Wnt-releasing Stromal 2 cell markers, specifically F3/Cd142, accompanied by an elevation in RSPO3-releasing Stromal 3 cell marker levels, and marked expansion of the pro-inflammatory Stromal 4 cell population (19, 42). These data indicate that Stromal 3 cells are upregulated exclusively in the aganglionic rectum, along with their products RSPO3. It has been reported that RSPO3 is significantly more potent than RSPO1 in maintaining crypt-derived intestinal stem cells and may be sufficient to potentiate Wnt signaling in the maintenance of intestinal homeostasis (57), where RSPO3 has also been shown to support intestinal organoid growth *in vitro* (60). Similarly, we did not observe any epithelial or regeneration defects in HSCR patient aganglionic rectum where RSPO3-releasing Stromal 3 is present. This suggests RSPO3 as a compensatory source of Wnt signaling in response to the decreased Stromal 2 cell population in this segment. An excessive presence of RSPO3 plays a critical role in the development of fibrosis in multiple organs (61), aligning with a recent observation that identified the presence of fibrosis in the aganglionic rectum of individuals with HSCR (27), which ultimately led to the formation of strictures. Conversely, where normal repair and regeneration responses mediated by crypt niche Stromal 2 are compromised in IBD, the expansion of pro-inflammatory Stromal 4 cells and their secreted factors prevent the resolution phase of a wound-healing response (19). We observed the presence of the inflammatory-related Stromal 4 cell population, not in the aganglionic rectum, but rather in the adjacent distal ganglionic colon. Moreover, we found that the Stromal 4 cells are absent in the proximal ganglionic colon (colostomy level) and instead present in the distal ganglionic colon (Fig. 6G). These findings indicate that the shift in stromal cells occurred in the colon proximal to the functional obstruction caused by aganglionosis, suggesting that this is a feature of HSCR. Stromal cells derived from this segment hindered the growth of epithelial organoids from control patients, suggesting that Stromal 4 cells in the distal colon contributed to the observed epithelial damage in HAEC. Inhibition of Stromal 4 has shown promise in attenuating DSS colitis and reducing circulating markers of oxidative stress (19). The prediction model for drug-cell interactions has identified a therapeutic candidate for suppressing Stromal 4, offering a potential complementary approach to using Stromal 4 inhibitors to mitigate bowel injury in human. Excitingly, administration of Ibuprofen through enema has been tested and optimized in pediatric patients with patent ductus arteriosus (62–64), suggesting that this local delivery approach could accelerate translation of our findings and enable drug repurposing for intestinal diseases. Activated Stromal 4 cells have emerged with the ability to mobilize the immune response and stimulate the formation of tertiary lymphoid follicles (19, 65). Further exploration of the emergence of Stromal 4 in the distal ganglionic colon of HSCR and its role in orchestrating the microenvironment in this segment is essential for a deeper understanding of the pathogenesis of HAEC. Moreover, employing Stromal 4 cell marker MMP1 staining at the edge of the bowel resected from HSCR patients can be an innovative approach. This method offers valuable guidance for surgical margin determination, facilitating a more precise and tailored surgical approach, while also serving as predictive biomarkers to gauge the likelihood of HAEC development. The *in vitro* stromal/organoid platform provides a valuable tool for evaluating various interventions in a controlled laboratory environment (66). Notably, we have not only identified Stromal 4 successfully but have also demonstrated the therapeutic potential of drugs specifically chosen to target and inhibit this cell population. This discovery holds significant promise for the treatment of HAEC, allowing further exploration and potential clinical applications.

The translation of these experimental findings into clinical practice presents an exciting challenge. It is important to highlight that although human organoids responded positively to the Stromal 4 inhibitor, this promising response has not yet been translated into improved animal survival or noticeable beneficial effects in mice *in vivo*. This presents an opportunity for further research and refinement, considering the disparities in stromal cells between humans and mice, as well as the unique challenges posed by the compromised intestinal health in mice due to HSCR. An additional area of consideration for potential drawbacks is the method of human colonic tissue collection in the present study. As obtaining colonic biopsies from age-matched normal controls is challenging, we chose to study children diagnosed with Hirschsprung allied disorders. These patients had constipation, gut dysmotility, normal ganglion cells in the rectum, were unresponsive to medical treatment and ultimately required colonic resection, making them ideal controls for the present study involving RNA sequencing. However, due to efforts to avoid surgical interventions, infants with Hirschsprung allied disorders were initially treated conservatively, explaining the difference in age between HSCR and controls. It is important to note that the colon of children with Hirschsprung allied disorders closely resemble normal healthy tissue, due to the presence of neurons, in contrast with the colon of HSCR patients which lacks neurons in the aganglionic rectum. Furthermore, to strengthen our comparative evidence, we evaluated the proximal ganglionic colon of patients with HSCR as additional internal controls. Although the initial single-cell RNA sequencing was conducted only in one centre, subsequent validations through different experiments were carried out independently at three centers (Toronto Canada, London UK, and Beijing China) encompassing a wider patient demographic, enhancing data reproducibility and scientific validity. The final limitation in the present study lies in the omission of microbiota analysis. This is particularly noteworthy as previous research has demonstrated the impact of microbiota on cellular regulatory mechanisms. Nevertheless, the approach in this investigation has yielded valuable insights into the current management of HSCR and HAEC, contributing to the ever-growing understanding of these conditions.

The present study unveiled the unique observation that alterations in epithelial regenerative capacity precede the onset of enterocolitis-like intestinal injury. This temporal sequence suggests that regenerative dysfunction may serve as a contributing factor or even a precursor to HAEC development, challenging the conventional understanding of HAEC primarily as a result of aganglionosis-induced physical obstruction. Remarkably, changes in stromal cells, including a reduction in Wnt-releasing Stromal 2 cells and the emergence of pro-inflammatory Stromal 4 cells, occur prior to HAEC onset. This indicates that stromal cell remodeling could act as a precondition in the distal ganglionic colon in HSCR, actively contributing to subsequent HAEC development. Understanding this pathogenic progression from Hirschsprung’s disease to Hirschsprung-associated enterocolitis holds significant implications for advancing early diagnostic and therapeutic strategies in great benefit of these patients.

## Materials And Methods

### Animal

All animal experiments were approved by the Animal Care Committee at The Hospital for Sick Children (#64987), and all methods were performed according to their guidelines and regulations. *Ednrb*^*-/-*^ mice were obtained from Juntendo University, Japan and established as previously reported (67, 68). Homozygous mutant mice were identified by their white coat color and confirmed by their genotypes. Mice were sacrificed at postnatal day 14 (P14) or P21 by CO_2_ asphyxiation. Control colonic tissues were collected from homozygous wildtype (WT) littermates. The colon was removed and preserved for further analysis.

*Lgr5-EGFP-IRES-creERT2* mice were obtained from Jackson Laboratory (Sacramento, CA), which allowed the visualization of LGR5+ ISC expression by genetically fusing the green fluorescence protein (GFP) with LGR5+ ISC (36). *Ednrb*^*-/-*^ mice were cross-bred with *Lgr5-EGFP-IRES-creERT2* mice to generate *Lgr5-EGFP; Ednrb*^*-/-*^ mice and were used to study the expression of LGR5+ ISC in HSCR mice colon.

### Human colonic tissue

Ethical approval for this study was obtained from the Research Ethics Board of The Hospital for Sick Children, Toronto, Canada (#1000056881); Capital Institute of Pediatrics, Beijing, China (#SHERLL 2019049); and Great Ormond Street Institute of Child Health, University College London, London, UK (#08ND13). Human colon samples were collected after informed consent in compliance with all relevant ethical regulations and the patients’ information presented in the Supplementary Table.

For single-cell RNA sequencing analysis, patients who received surgery for short segment HSCR (n=5) at the Department of General Surgery, Capital Institute of Pediatrics Affiliated Children’s Hospital were recruited. The clinical diagnosis for each segment was confirmed through both intraoperative frozen pathological examination and postoperative immunohistochemical staining. Additionally, samples from two patients from this center were regarded as non-HSCR control (n=2). These individuals, despite experiencing gut motility dysfunction and retaining ganglion cells while being unresponsive to alternative treatments, required surgical bowel resection, making them suitable candidates for this study. To minimize potential sources of variability unrelated to biology and variations in luminal content exposure, full-thickness biopsy tissue from the same patients were obtained. Tissue from the aganglionic rectum (HSCR_R) and the distal ganglionic colon (HSCR_D) from HSCR patients, as well as from the rectum (Control_R) and the distal colon (Control_D) from non-HSCR patients were analyzed.

Pathological sections of human HSCR colon were obtained from the pathology departments at the above centers. The diagnoses of HSCR were verified through intraoperative frozen sections, histologic hematoxylin and eosin (H&E) examination, and postoperative immunohistochemical staining. According to the morphology of the colon, specimens were divided into 3 segments: aganglionic rectum, distal ganglionic colon, and proximal ganglionic colon. Although operations were performed on HSCR patients without active HAEC symptoms, but pathological examination of the resected tissue revealed epithelial damage in the specimens obtained from HSCR pull-through surgery. Based on the histopathologic assessment of epithelial damage severity, the samples were categorized into two groups: HSCR patients without HAEC, and HAEC patients. The severity of enterocolitis was scored using an established histopathologic scoring system (69). The distal colon and rectum tissue, resected for reasons other than HSCR, were used as non-HSCR controls in this study. Moreover, tissue was obtained from above the aganglionic segment in patients with a colostomy during the HSCR pull-through operation.

In addition, human intestinal organoids and stromal cells were derived from distal ganglionic colon of HSCR patients and non-HSCR control patients from Capital Institute of Pediatrics, Beijing, China and Great Ormond Street Institute of Child Health, University College London, London, UK, after informed consent.

### Histological staining and assessment

Mice colonic tissues embedded in paraffin were cross-sectioned (5µm) and stained with hematoxylin and eosin. Histological sections were assessed by three blinded investigators following an established histopathological scoring system (70), as grade 0 = no damage; grade 1 = epithelial cell lifting or separation; grade 2 = sloughing of epithelial cells to the mid-crypts level; grade 3 = necrosis of the entire crypts; grade 4 = transmural necrosis.

### Immunostaining

Sections of colonic tissue were incubated with 1 in 500 dilutions of primary antibodies (Table S1) overnight at 4 °C. For immunofluorescence staining, sections were then incubated with 1 in 1000 diluted secondary antibodies (Table S1), and DAPI for visualization of cell nuclei (Vector Laboratories, Burlington, ON), at room temperature for 1 hour. For immunohistochemistry staining, sections were incubated with 1 in 1000 diluted HRP-conjugated secondary antibodies, followed by streptavidin–biotin complex peroxidase kit (LASB + Kit, Dako, Denmark) and hematoxylin counterstaining. Slides were imaged using a Nikon TE-2000 digital microscope equipped with a Hamamatsu C4742-80-12AG camera. Three blinded investigators counted the number of positively labeled cells from at least ten crypts of the intestine and five images from each subject.

### Gene quantification

RNA was isolated from colonic tissue with TRIzol (Invitrogen). Total RNA (1µg) was reverse transcribed using qScript cDNA SuperMix (Quanta Biosciences, Gaithersburg). SYBR green-based quantitative polymerase chain reaction (qPCR) was performed using a CFX384 C1000 Thermal Cycler (Bio-Rad) and Advanced qPCR MasterMix (Wisent, Quebec) using manufacturer’s protocol and primers listed in Table S1. Data was analyzed using CFX Manager 3.1 (Bio-Rad). Results are from three independent experiments performed in triplicate. Expression levels were calculated by the ΔΔCt method and normalized to reference housekeeping gene *glyceraldehyde 3-phosphate dehydrogenase (Gapdh)* (71).

### Single-molecule fluorescence in situ hybridization (smFISH)

smFISH and the quantification were performed according to the manufacturer’s protocol described in the RNAscope Multiplex Fluorescent Detection Kit v2 (323120, ACDBio). The RNAscope Probes (ACDBio)-Mm-Lgr5 (Cat No. 312171) and Mm-Wnt5a (Cat No. 316791) were used for smFISH. Images were taken with a Nikon A1R Confocal microscope. Quantification was performed according to the manufacturer’s protocol by three blinded investigators.

### Protein quantification

Protein expression was quantified using immunoblotting analysis as previously described (71). The membrane was probed with primary antibodies (1:500) overnight at 4 °C and secondary antibodies (1:1000) at room temperature. Immuno-positive bands were detected using an ECL Plus kit (Invitrogen, Carlsbad, CA). Band intensities were determined using an Odyssey scanner (LI-COR Biosciences, Lincoln, NE). Densitometry ratios were calculated relative to levels of loading control.

### Flow cytometry

Flow cytometry was performed to confirm the presence of MMP1 expression in the stromal cells. Briefly, stromal cells were gentle permilized and incubated with MMP1 antibody at 4°C overnight and were then incubated with FITC secondary antibody for 2 hours at room temperature, washed and analyzed by flow cytometry on a Beckman Coulter Gallios flow cytometer. Data was analyzed using FlowJo software. Mean fluorescence intensity was calcuted and presented for the intracellular expression of the stromal 4 MMP1.

### Mouse organoids

Intestinal organoids were cultured according to protocols previously described (72). Colonic tissues were harvested and cut into 1-2mm small segments. Colonic crypts were isolated by digestion with Gentle Cell Dissociation Reagent (StemCell Technologies, Cambridge, MA) for 15 minutes and pelleted by centrifugation. Crypts were then re-suspended in Matrigel (Corning, New York) and transferred into 24-well plates. After polymerization, mouse IntestiCult organoid growth medium (StemCell Technologies, Cambridge, MA) supplemented with penicillin-streptomycin (100U/mL) was overlaid on the gel in each well. Organoids were maintained in a 37°C and 5% CO_2_ incubator with the culture medium replaced every 48 hours. Organoids were imaged daily, and their surface area was calculated using Image J software 1.53e.

### Human colonic organoids and stromal cells co-culture

Human colon organoids were cultured as described previously (66, 72), with human IntestiCult organoid growth medium (StemCell Technologies, Cambridge, MA) and 10 μM Y-27632 (StemCell Technologies, Cambridge, MA), a ROCK inhibitor added to the medium for primary culture. Cell proliferation within organoids was assessed using the BeyoClick™ EdU Cell Proliferation Kit with Alexa Fluor 488 (Beyotime, C0071S). Apoptotic characteristics of organoid cells were evaluated with the One-Step TUNEL Apoptosis Assay Kit (Beyotime, C1089).

Stromal cells were cultured in DMEM medium with 10% heat-inactivated fetal bovine serum (FBS) (vol/vol), penicillin–streptomycin (100U/mL) and 1× insulin–transferrin–selenium A, according to the published protocol (66). Ibuprofen (100ug/mL, Sigma #14883) was added in the culture medium of stromal cells. Approximately 25-40 organoids and 5x10^4^ stromal cells derived from HSCR patients or non-HSCR control patients were re-suspended in Matrigel (Corning, New York), transferred into 24-well plates (40 μL/well), cultured in organoid media and maintained in a 37 °C incubator for 5 days (59). The co-culture was imaged daily.

The supernatant samples from stromal cells treated with or without Ibuprofen were analysed using Luminex liquid suspension chip assays at Wayen Biotechnologies (Shanghai, China). The levels of 48 cytokines were determined using the Bio-Plex Pro Human Cytokine Screening 48-plex Panel (12007283; Bio-Rad Laboratories, Hercules, CA) according to the manufacturer’s instructions. Detection results were obtained after analysis on the Bio-Plex instrument.

To assess the assembly potential of free-floating organoids, HSCR organoids were cultured in a collagen I hydrogel, enabling them to organize into complex assembloids with a polarized epithelium and a shared lumen, as demonstrated in previous studies (73, 74).

### Human tissue dissociation, single-cell suspension preparation and sequencing

The colon tissue preserved in GEXSCOPE Tissue Preservation Solution (Singleron) and per Singleron lab’s standard protocol. After digestion using 2 mL of GEXSCOPE Tissue Dissociation Solution (Singleron), the cell suspensions were filtered through a 40-micron sterile strainer (Falcon, Cat. No. 352340) and centrifuged. The resulting pellets were resuspended in 1 mL of PBS. Red blood cells were eliminated by adding 2 mL of RBC lysis buffer (Roche, Cat No. 11814389001), followed by another round of centrifugation and resuspension. Cell viability was verified using trypan blue staining (Bio–Rad, Cat No. 1450013) under a Nikon ECLIPSE Ts2 microscope, ensuring a concentration of 100,000 cells/mL and viability exceeding 80%. scRNA-seq libraries were prepared using the GEXSCOPE Single-Cell RNA Library Kit (Singleron Biotechnologies), which entailed cell lysis, mRNA trapping, cell labeling with barcodes, and marking mRNA with UMIs, followed by reverse transcription of the mRNA into cDNA, amplification, and fragment capture. Sequencing was performed on an Illumina HiSeq X, generating 150 bp paired end reads.

### Single-cell RNA-seq preprocessing

After quality control, single cell sequencing data were aligned to the GRCh38 human reference genome and quantified using Cell Ranger (version 6.1.2, 10x Genomics Inc). The preliminary filtered data generated from Cell Ranger underwent further filtering: cells with UMI counts below 30,000, gene counts between 200 and 5,000, and those with over 50% mitochondrial content were removed. To remove the potential doublets, Scrublet was used for each sequencing library with the expected doublet rate set to be 0.12. For dimensionality reduction, clustering, and data normalization, Seurat v4 functions was performed (75). Specifically, data normalization and scaling were achieved with the “NormalizeData()” and “ScaleData()” functions. The top 2,000 variable genes were identified using “FindVariableFeatures()” for Principal Component Analysis (PCA). When integrating multiple samples, a CCA-based workflow in Seurat was implemented. Louvain community detection, applied to the batch-corrected principal components via Seurat, facilitated clustering. The top 20 principal components were used as input for Louvain clustering using the FindClusters() function at resolution 1.0. Lastly, the visualization of single-cell transcriptional profiles and clusters was executed through Uniform Manifold Approximation and Projection (UMAP) using in Seurat’s RunUMAP() function with these parameters (n_neighbors = 30L, min_dist = 0.5).

### Cell type annotation

A semi-automatic annotation approach was employed for cell type classification, encompassing two clustering rounds to discern major and minor cell types. Annotations ascribed were based on major and minor cell types previously published(39). The K-nearest neighbour (KNN) algorithm, implemented through the RunKNNPredict() function in the R SCP package, facilitated cell type predictions. The initial unsupervised clustering identified eight major cell types: T cells, myeloid cells, B/plasma cells, neuronal cells, stromal cells, endothelial cells, fibroblasts/pericytes, and epithelial cells. Subsequent clustering specifically targeted stromal and epithelial cells to identify minor cell types. The protocol of secondary clustering used was identical to the first-round clustering, with a resolution spanning 0.8 to 1.5. Minor cell types were manually annotated, relying on specific markers within each subcluster, the predictions of minor cell types and markers reported in the literature.

### Estimation of Cell Cycle Status and Module Score Definition

To assess the proliferation status of individual cells, evaluation was based on a characteristic gene set involved in the cell cycle. This included 43 G1/S and 54 G2/M cell cycle genes, as previously described (76). Cells manifesting high G1/S or G2/M scores were categorized as cycling, while those with low scores in both G1/S and G2/M were designated as non-cycling. A data-derived threshold, set at 2 MADs (Median Absolute Deviations) above the median, was applied to differentiate high from low scores. The AddModuleScore() function from the Seurat R package was employed to ascertain the extent to which individual cells expressed specific predefined expression programs. Gene signatures were acquired from The Molecular Signatures Database (MSigDB) (https://www.gsea-msigdb.org/gsea/msigdb). Pathway signature scores were generated by utilizing the ScoreSignatures_UCell() function from the UCell package (77).

### Differential Gene Expression Analysis

Differentially Expressed Genes (DEGs) within a specific cell type, in comparison to all other cell types, were identified using the FindAllMarkers() function from the Seurat package, relying on a one-tailed Wilcoxon rank sum test. Adjustments for multiple testing were made using the Bonferroni correction. For DEG computation, genes were considered if they were expressed in at least 25% of cells in either of the two compared populations, and if the expression difference on a natural logarithm scale exceeded 0.25. Gene Set Enrichment Analysis (GSEA) was conducted using the R package clusterProfiler (78).

### Single-cell Trajectory Analysis with Monocle v.2

Monocle2 (79) was employed to delineate cell state transitions in total epithelial cells. This software employs a reverse graph embedding method to map single-cell trajectories. The analysis was initialized using UMI count matrices, adopting the default setting and the negbinomial.size() parameter to formulate a CellDataSet object. For the trajectory of normal epithelial cells, epithelial differentiation marker genes, as highlighted in (80), facilitated semi-supervised trajectory reconstruction. Both dimensional reduction and cell ordering were executed via the DDRTree method and the orderCells() function, designating stem cells as the pseudotime analysis starting point. The HSCR and non-HSCR control groups were processed independently. To synchronize the trajectories of the HSCR and control groups, dynamic time warping was applied as outlined in (81), ensuring alignment to a unified pseudotime axis. To pinpoint genes marking differences in the interplay between pseudotime and disease condition across both trajectories, differential gene expression analysis was conducted using a comprehensive model of ‘y ~ pseudotime*condition’ and a simplified model of ‘y ~ pseudotime’.

### Cell–Cell Interaction (CCI) Analysis

Prediction of cell–cell interactions across all cell types was performed utilizing known ligand–receptor pairs with the help of LIANA (82). The liana_wrap() and liana_aggregate() functions allowed for the adoption of default parameters to obtain consensus ligand-receptor pairs derived from various methods.

### PHATE Assessment

PHATE (Potential of Heat-diffusion for Affinity-based Trajectory Embedding) was selected as an alternative dimensionality reduction technique to effectively capture the global structures in biological systems, particularly those with critical developmental trajectories (83). For the PHATE calculation, the first 20 Principal Components (PCs) were used as inputs. In terms of RNA velocity analysis, loom files were created from the output of Cell Ranger, utilizing the velocyto Python package (with the GRCh38 as the reference genome) (84). The analysis of RNA velocity was conducted using the scVelo Python package, applying its default settings (85).

### Tissue distribution of clusters

The ratio of observed-to-expected cell number Ro/e for each cluster was calculated, as previously described (86), which allowed for the quantification of tissue preference for each cluster in different locations. The chi-squared test was utilized to get the expected cell numbers for each combination of cell clusters and tissues. If Ro/e > 1, one cluster was identified as being enriched in a specific tissue. If Ro/e < 1, one cluster was identified as being depleted in a specific tissue.

### Targeted Drug Prediction in Single-Cell Analysis

To predict targeted drugs in single-cell analysis, the drug2cell tool was used to merge drug–target interactions from the ChEMBL database with user-input single-cell data to thoroughly assess drug-target expression at the single-cell level (87). Different drugs for specific cell types in comparison to all other cell types were identified using the FindAllMarkers() function from the Seurat package. To pinpoint highly specific drugs for the “Stromal 4” cell type, drugs were ranked based on their adjusted p-values, and a filter was applied to retain those with a percentage value above 0.9.

### Statistics

All analyses were performed with GraphPad prism 6. All results were performed at least in triplicate and expressed as mean ± SEM. P-values were determined using nonparametric unpaired Student’s t-tests (Mann–Whitney U test) or using one-way ANOVA with post hoc Turkey test as appropriate. P <0.05 was considered as statistically significant.

### Data availability

All relevant data supporting the key findings of this study, as well as the raw image data generated in this study, are available within the paper and its Supplementary Information or from the corresponding author upon reasonable request. The raw sequence data reported in this paper have been deposited in the Genome Sequence Archive (88) of the National Genomics Data Center (89), China National Center for Bioinformation / Beijing Institute of Genomics, Chinese Academy of Sciences (GSA-Human: HRA004266) that are publicly accessible at https://ngdc.cncb.ac.cn/gsa-human.

## Supplementary Material

data file S1

data file S2

data file S3

data file S4

data file S5

data file S6

Fig. S1

Supplementary Information

## Data Availability

All relevant data supporting the key findings of this study, as well as the raw image data generated in this study, are available within the paper and its Supplementary Information or from the corresponding author upon reasonable request. The raw sequence data reported in this paper have been deposited in the Genome Sequence Archive of the National Genomics Data Center, China National Center for Bioinformation / Beijing Institute of Genomics, Chinese Academy of Sciences (GSA-Human: HRA004266) that are publicly accessible at https://ngdc.cncb.ac.cn/gsa-human.

## One Sentence Summary

The impaired stromal-epithelium crosstalk in Hirschsprung’s disease highlights the risk of epithelial damage, suggesting a therapeutic option.
