# Supplementary material for: Impairment of stromal-epithelial regenerative cross-talk in Hirschsprung disease primes for the progression to enterocolitis
Source: Sci Transl Med. Author manuscript; Available in PMC 2025 Nov 10. (PMC7618341; doi:10.1126/scitranslmed.adp4679)
Supplement: Fig. S1 [file EMS210296-supplement-Fig__S1.pdf]

Science Translational Medicine
AAAS

Supplementary Materials for

Impairment of stromal-epithelial regenerative cross-talk in Hirschsprung disease primes for the progression to enterocolitis

Zhen Zhang *et al.*

Corresponding author: Qian Jiang, teaco@126.com; Agostino Pierro, agostino.pierro@sickkids.ca; Bo Li, bo.li@sickkids.ca

Sci. Transl. Med. **17**, eadp4679 (2025)
DOI: 10.1126/scitranslmed.adp4679

The PDF file includes:

Materials and methods
Figs. S1 to S8
Tables S1 to S6
Legends for data files S1 to S6
References (*61–76*)

Other Supplementary Material for this manuscript includes the following:

Data files S1 to S6
MDAR Reproducibility Checklist

SUPPLEMENTARY MATERIALS AND METHODS

Histological staining and assessment

Human and mice colonic tissues were embedded in paraffin, cross-sectioned (5µm) and stained with hematoxylin and eosin. Histological injury grading scores were given to each sample as the median from the assessment by three blinded investigators following an established histopathological scoring system (*57, 61*), as grade 0 = no damage; grade 1 = epithelial cell lifting or separation; grade 2 = sloughing of epithelial cells up to the mid-crypts; grade 3 = necrosis of the entire crypts; grade 4 = transmural necrosis.

Immunostaining

Sections of colonic tissue were incubated with 1 in 500 dilutions of primary antibodies overnight at 4 °C. For immunofluorescence staining, sections were then incubated with 1 in 1000 diluted secondary antibodies and DAPI for visualization of cell nuclei (Vector Laboratories), at room temperature for 1 hour. For immunohistochemistry staining, sections were incubated with 1 in 1000 diluted HRP-conjugated secondary antibodies, followed by streptavidin–biotin complex peroxidase kit (LASB + Kit, Dako) and hematoxylin counterstaining. Slides were imaged using a Nikon TE-2000 digital microscope equipped with a Hamamatsu C4742-80-12AG camera. Three blinded investigators counted the number of positively labeled cells from at least ten crypts of the intestine and three images from each patient. All primary and secondary antibodies are listed in **Table S5.**

Gene quantification

RNA was isolated from colonic tissue with TRIzol (Invitrogen). Total RNA (1µg) was reverse transcribed using qScript cDNA SuperMix (Quanta Biosciences). SYBR green-based real time-quantitative polymerase chain reaction (RT-qPCR) was performed using a CFX384 C1000 Thermal Cycler (Bio-Rad) and Advanced qPCR MasterMix (Wisent) using the manufacturer's protocol and the primers listed in **Table S6**. Data was analyzed using CFX Manager 3.1 (Bio-Rad). Results are from three independent experiments performed in triplicate. Expression was calculated by the ΔΔCt method and normalized to reference housekeeping gene *glyceraldehyde 3-phosphate dehydrogenase (Gapdh)* (*62*).

Single-molecule fluorescence in situ hybridization (smFISH)

smFISH was performed according to the manufacturer's protocol described in the RNAscope Multiplex Fluorescent Detection Kit v2 (323120, ACDBio). The RNAscope Probes (ACDBio)-Mm-Lgr5 (Cat No. 312171) and Mm-Wnt5a (Cat No. 316791) were used for smFISH. Images were taken with a Nikon A1R Confocal microscope. Quantification was also performed according to the manufacturer's protocol by three blinded investigators.

Protein quantification

Protein expression was quantified using immunoblotting analysis as previously described (*62*). The membrane was probed with primary antibodies (1:500) overnight at 4 °C and secondary antibodies (1:1000) at room temperature. Immuno-positive bands were detected using an ECL Plus kit (Invitrogen, Carlsbad, CA). Band intensities were determined using an Odyssey FC scanner (LI-COR Biosciences). Densitometry ratios were calculated relative to the loading control.

Flow cytometry

Flow cytometry was performed to confirm the presence of MMP1 in HSCR stromal cells. Briefly, stromal cells were gently permeabilized and incubated with MMP1 antibody at 4°C overnight and were then incubated with FITC secondary antibody for 2 hours at room temperature, washed and analyzed by flow cytometry on a Beckman Coulter Gallios flow cytometer. Data was analyzed using FlowJo software. Mean fluorescence intensity was calculated and used to plot MMP1 expression.

Mouse organoids

Intestinal organoids were cultured according to protocols previously described (*63*). Colonic tissues were harvested and cut into 1-2mm small segments. Colonic crypts were isolated by digestion with Gentle Cell Dissociation Reagent (STEMCELL Technologies) for 15 minutes and pelleted by centrifugation. Crypts were then re-suspended in Matrigel (Corning) and transferred into 24-well plates. After polymerization, mouse IntestiCult organoid growth medium (STEMCELL Technologies) supplemented with penicillin-streptomycin (100U/mL) was overlaid on the gel in each well. Organoids were maintained in a 37°C and 5% CO_2 incubator with the culture medium replaced every 48 hours. Organoids were imaged daily, and their surface area was calculated using Image J software 1.53e.

Co-culture of human colonic organoids and stromal cells

Human colon organoids were cultured as described previously (*53, 63*), with human IntestiCult organoid growth medium (STEMCELL Technologies) and 10 µM Y-27632 (STEMCELL Technologies), a ROCK inhibitor added to the medium for primary culture. Cell proliferation within organoids was assessed using the KI67 (Cell Signaling Technology, #9129) staining, and apoptotic characteristics of organoid cells were evaluated with cleaved caspase 3 (Cell Signaling Technology, #9661) staining.

Stromal cells were cultured in DMEM medium with 10% heat-inactivated fetal bovine serum (FBS) (vol/vol), penicillin–streptomycin (100U/mL) and 1× insulin–transferrin–selenium A, according to the published protocol (*53*). Ibuprofen (100ug/mL, Sigma-Aldrich #14883) was added in the culture medium of stromal cells. About 25-40 organoids and $5x10^4$ stromal cells derived from HSCR or control colons were re-suspended in Matrigel (Corning), transferred into 24-well plates (40 µL/well), cultured in organoid media and maintained in a 37 °C incubator for 5 days (*64*). The co-culture was imaged daily.

The supernatant samples from stromal cells treated with or without ibuprofen were analysed using Luminex liquid suspension chip assays at Wayen Biotechnologies. The expression of 48 cytokines were determined using the Bio-Plex Pro Human Cytokine Screening 48-plex Panel (Bio-Rad Laboratories #12007283) according to the manufacturer's instructions. Detection results were obtained after analysis on the Bio-Plex instrument.

To assess the assembly potential of free-floating organoids, HSCR organoids were cultured in a collagen I hydrogel, enabling them to organize into complex assembloids with a polarized epithelium and a shared lumen, as demonstrated in previous studies *(65, 66)*.

Human tissue dissociation, single-cell suspension preparation and sequencing

The colon tissue preserved in GEXSCOPE Tissue Preservation Solution (Singleron) and per Singleron's standard protocol. After digestion using 2 mL of GEXSCOPE Tissue Dissociation

Solution (Singleron), the cell suspensions were filtered through a 40-micron sterile strainer (Falcon, Cat. No. 352340) and centrifuged. The resulting pellets were resuspended in 1 mL of PBS. Red blood cells were eliminated by adding 2 mL of RBC lysis buffer (Roche, Cat No. 11814389001), followed by another round of centrifugation and resuspension. Cell viability was verified using trypan blue staining (Bio–Rad, Cat No. 1450013) under a Nikon ECLIPSE Ts2 microscope, ensuring a concentration of 100,000 cells/mL and viability exceeding 80%. scRNA-seq libraries were prepared using the GEXSCOPE Single-Cell RNA Library Kit (Singleron Biotechnologies), which entailed cell lysis, mRNA trapping, cell labeling with barcodes, and marking mRNA with UMIs, followed by reverse transcription of the mRNA into cDNA, amplification, and fragment capture. Sequencing was performed on an Illumina HiSeq X, generating 150 bp paired end reads.

Single-cell RNA-seq preprocessing

After quality control, single cell sequencing data were aligned to the GRCh38-2020-A human reference genome and quantified using Cell Ranger (version 6.1.2, 10x Genomics Inc). The preliminary filtered data generated from Cell Ranger underwent further filtering: cells with UMI counts below 30,000, gene counts between 200 and 5,000, and those with over 50% mitochondrial content were removed. To remove the potential doublets, Scrublet was used for each sequencing library with the expected doublet rate set to be 0.12. For dimensionality reduction, clustering, and data normalization, Seurat v4 functions was performed (*67*). Specifically, data normalization and scaling were achieved with the "NormalizeData()" and "ScaleData()" functions. The top 2,000 variable genes were identified using "FindVariableFeatures()" for Principal Component Analysis (PCA). When integrating multiple samples, a CCA-based workflow in Seurat was implemented. Louvain community detection, applied to the batch-corrected principal components via Seurat, facilitated clustering. The top 20 principal components were used as input for Louvain clustering using the FindClusters() function at resolution 1.0. Lastly, the visualization of single-cell transcriptional profiles and clusters was executed through Uniform Manifold Approximation and Projection (UMAP) using in Seurat's RunUMAP() function with these parameters (n_neighbors = 30L, min_dist = 0.5).

Cell type annotation

A semi-automatic annotation approach was employed for cell type classification, encompassing two clustering rounds to discern major and minor cell types. Annotations ascribed were based on major and minor cell types previously published (*69*). The K-nearest neighbour (KNN) algorithm, implemented through the RunKNNPredict() function in the R scp package, facilitated cell type predictions. The initial unsupervised clustering identified eight major cell types: T cells, myeloid cells, B/plasma cells, neuronal cells, stromal cells, endothelial cells, fibroblasts/pericytes, and epithelial cells. Subsequent clustering specifically targeted stromal and epithelial cells to identify minor cell types. The protocol of secondary clustering used was identical to the first-round clustering, with a resolution spanning 0.8 to 1.5. Minor cell types were manually annotated, relying on specific markers within each subcluster, the predictions of minor cell types and markers reported in the literature.

Estimation of cell cycle status and module score definition

To assess the proliferation status of individual cells, evaluation was based on a characteristic gene set involved in the cell cycle. This included 43 G1/S and 54 G2/M cell cycle genes, as previously described (*68*). Cells manifesting high G1/S or G2/M scores were categorized

as cycling, while those with low scores in both G1/S and G2/M were designated as non-cycling. A data-derived threshold, set at 2 MADs (Median Absolute Deviations) above the median, was applied to differentiate high from low scores. The AddModuleScore() function from the Seurat R package was employed to ascertain the extent to which individual cells expressed specific predefined expression programs. Gene signatures were acquired from The Molecular Signatures Database (MSigDB) (https://www.gsea-msigdb.org/gsea/msigdb). Pathway signature scores were generated by utilizing the ScoreSignatures_UCell() function from the UCell package (*69*).

Differential gene expression analysis

Differentially Expressed Genes (DEGs) within a specific cell type, in comparison to all other cell types, were identified using the FindAllMarkers() function from the Seurat package, relying on a one-tailed Wilcoxon rank sum test. Adjustments for multiple testing were made using the Bonferroni correction. For DEG computation, genes were considered if they were expressed in at least 25% of cells in either of the two compared populations, and if the expression difference on a natural logarithm scale exceeded 0.25. Gene set enrichment analysis (GSEA) was conducted using the R package clusterProfiler (*70*).

Single-cell trajectory analysis with Monocle v.2

Monocle2 (*36*) was employed to delineate cell state transitions in total epithelial cells. This software employs a reverse graph embedding method to map single-cell trajectories. The analysis was initialized using UMI count matrices, adopting the default setting and the negbinomial.size() parameter to formulate a CellDataSet object. For the trajectory of normal epithelial cells, epithelial differentiation marker genes, as highlighted in (*71*), facilitated semi-supervised trajectory reconstruction. Both dimensional reduction and cell ordering were executed via the DDRTree method and the orderCells() function, designating stem cells as the pseudotime analysis starting point. The HSCR and control groups were processed independently. To synchronize the trajectories of the HSCR and control groups, dynamic time warping was applied as outlined in (*37*), ensuring alignment to a unified pseudotime axis. To pinpoint genes marking differences in the interplay between pseudotime and disease condition across both trajectories, differential gene expression analysis was conducted using a comprehensive model of 'y ~ pseudotime*condition' and a simplified model of 'y ~ pseudotime'.

Cell–cell interaction (CCI) analysis

Prediction of cell–cell interactions across all cell types was performed utilizing known ligand–receptor pairs with the help of LIANA (*72*). The liana_wrap() and liana_aggregate() functions allowed for the adoption of default parameters to obtain consensus ligand-receptor pairs derived from various methods.

PHATE assessment

PHATE (Potential of Heat-diffusion for Affinity-based Trajectory Embedding) was selected as an alternative dimensionality reduction technique to effectively capture the global structures in biological systems, particularly those with critical developmental trajectories (*39*). For the PHATE calculation, the first 20 Principal Components (PCs) were used as inputs. In terms of RNA velocity analysis, loom files were created from the output of Cell Ranger, utilizing the velocyto Python package (with the GRCh38 as the reference genome) (*73*). The analysis of RNA velocity was conducted using the scVelo Python package, applying its default settings (*74*).

Tissue distribution of clusters

The ratio of observed-to-expected cell number ($R_{o/e}$) for each cluster was calculated, as previously described (*75*), which allowed for the quantification of tissue preference for each cluster in different locations. The chi-squared test was utilized to get the expected cell numbers for each combination of cell clusters and tissues. If Ro/e > 1, one cluster was identified as being enriched in a specific tissue. If Ro/e < 1, one cluster was identified as being depleted in a specific tissue.

Targeted drug prediction in single-cell analysis

To predict targeted drugs in single-cell analysis, the drug2cell tool was used to merge drug–target interactions from the ChEMBL database with user-input single-cell data to thoroughly assess drug-target expression at the single-cell resolution (*76*). Different drugs for specific cell types in comparison to all other cell types were identified using the FindAllMarkers() function from the Seurat package. To pinpoint highly specific drugs for the "Stromal 4" cell type, drugs were ranked based on their adjusted p-values, and a filter was applied to retain those with a percentage value above 0.9. Wilcoxon rank sum test was performed to acquire the false discovery rate adjusted p-value.

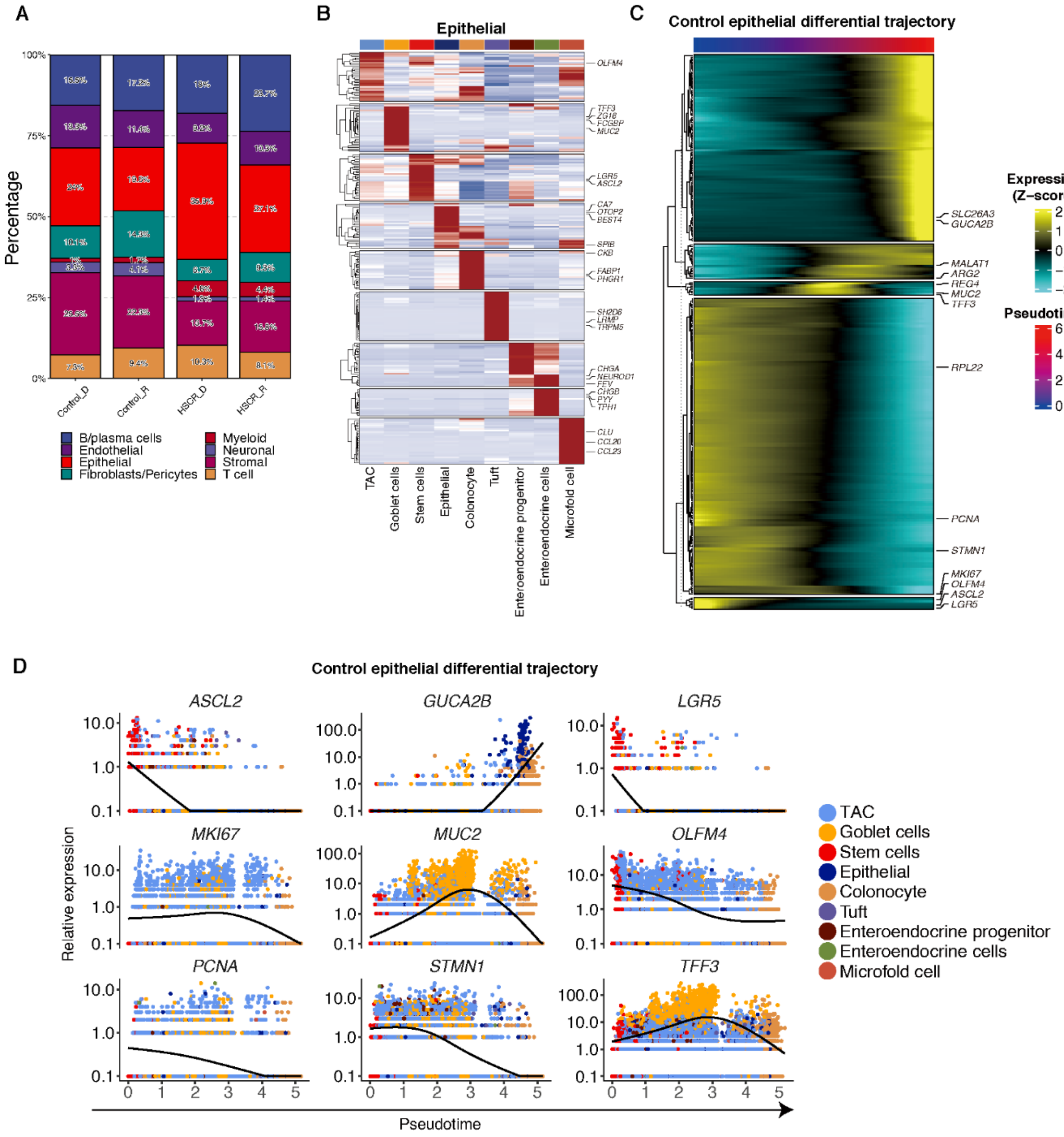

Fig. S1: Epithelial cells compartment across pseudotime.

(A) Proportion of all major cell subtypes in control and HSCR samples. **(B)** Heatmap visualizing marker genes for epithelial compartment populations. **(C)** Heatmap visualizing marker genes for epithelial trajectories. **(D)** Selected gene expression across epithelial differentiation pseudotime of control patients.

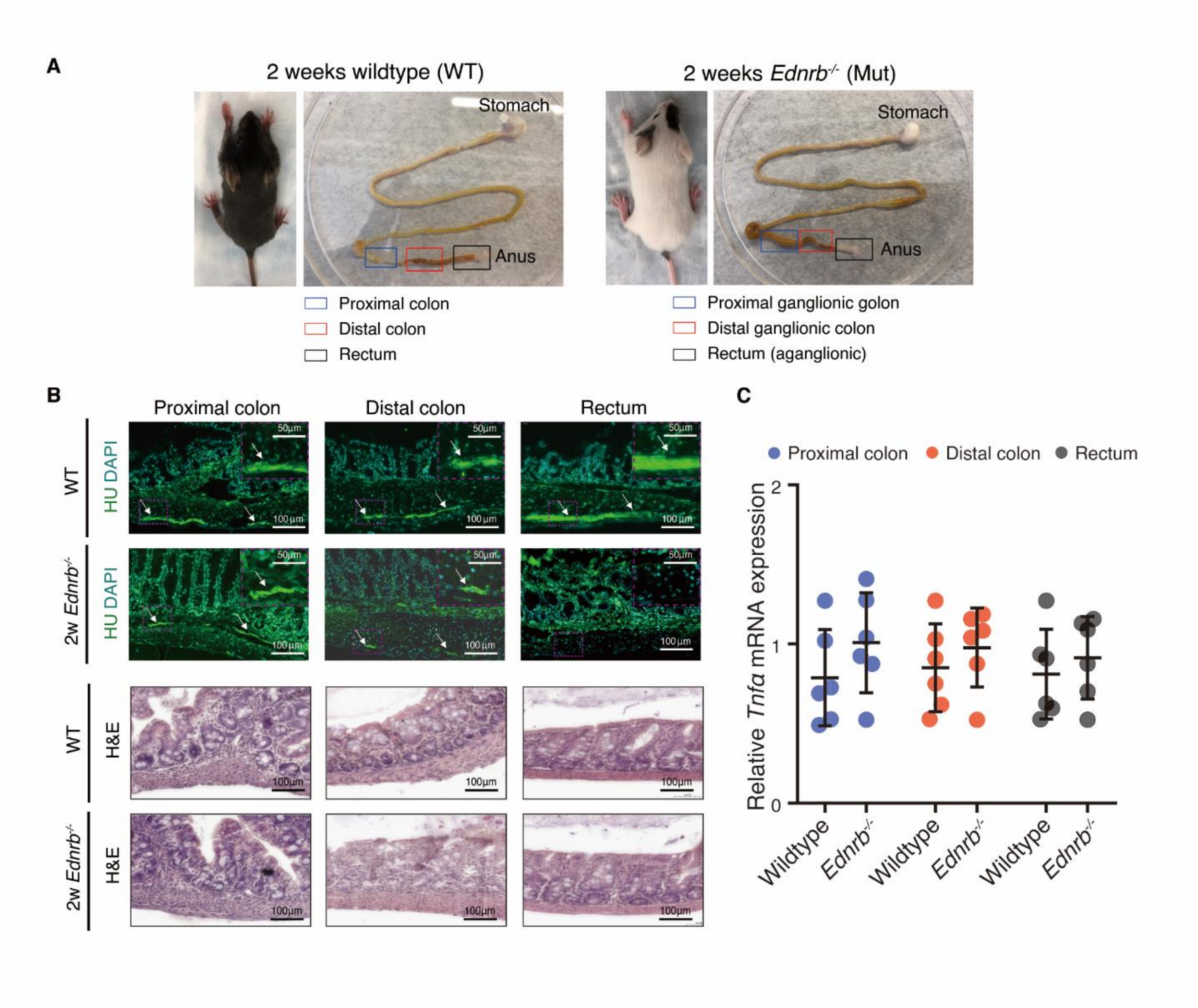

Fig. S2: Biometrics for 2-week-old *Ednrb*$^{-/-}$ mice.

(A) Representative images of dissected mouse whole gastrointestinal tract of wildtype (WT) and *Ednrb*$^{-/-}$ mice at 2 weeks of age, respectively. Blue box: proximal colon, red box: dilated distal ganglionic colon; and black box: aganglionic rectum; and their corresponding segments in wildtype colon. **(B)** Representative immunofluorescence micrographs of neuronal marker HU in the proximal colon, distal colon and rectum from 2-week-old wildtype and *Ednrb*$^{-/-}$ mice, white arrows indicate HU positive staining, with DAPI counterstaining of nuclei (blue) and representative H&E-stained histomicrographs of the proximal colon, distal colon and rectum from 2-week-old wildtype and *Ednrb*$^{-/-}$ mice. **(C)** mRNA expression of pro-inflammatory marker gene *Tnfa* in the proximal colon, distal colon and rectum of 2-week-old wildtype and *Ednrb*$^{-/-}$ mice. Scale bars for all images = 100µm, inset = 50µm. Wildtype mice n=6 and *Ednrb*$^{-/-}$ mice n=6. Experiments were repeated independently 3 times, with similar results. Each dot represents the average value of each individual. Data are presented as mean ± SD and compared using one-way ANOVA.

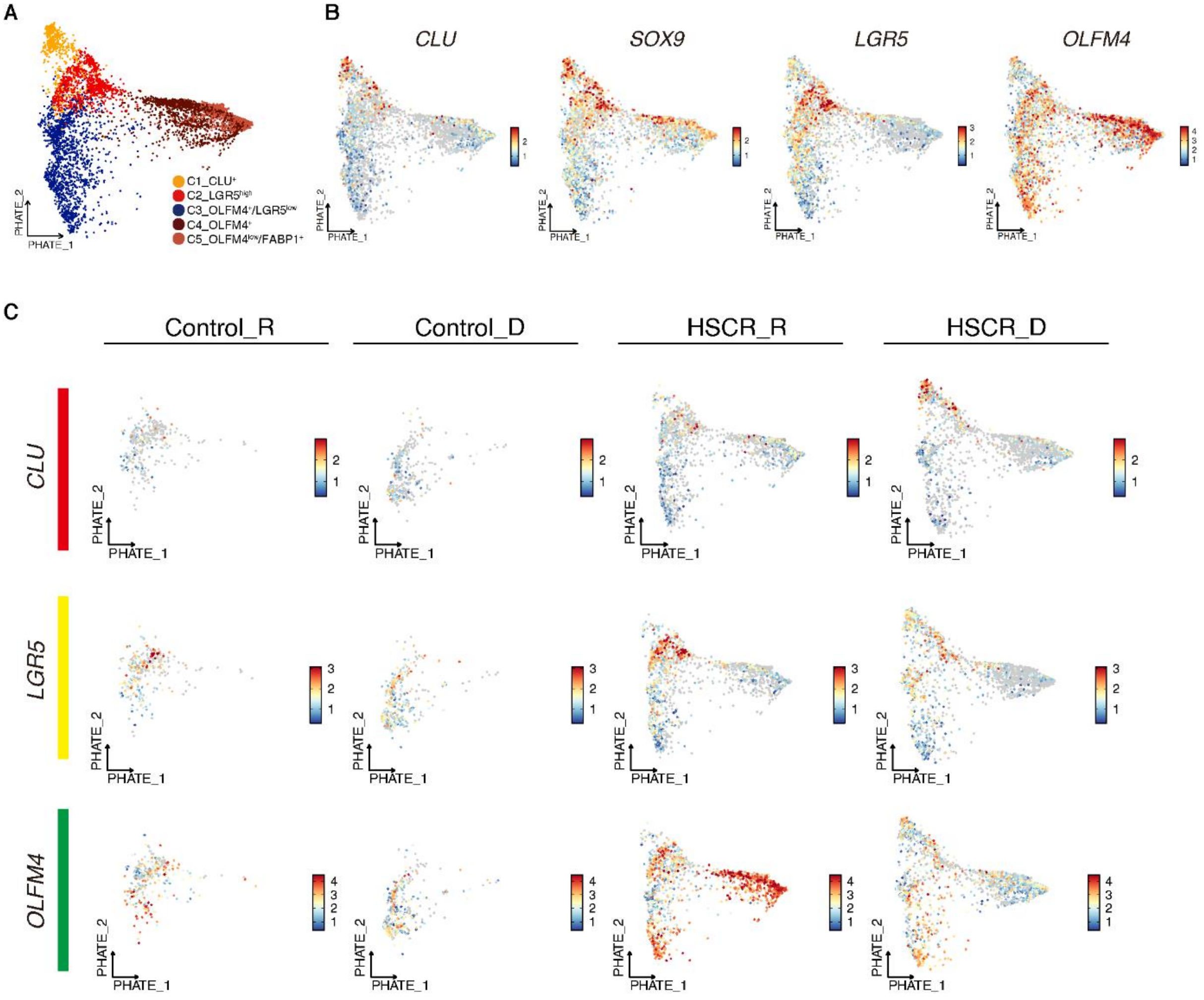

Fig. S3: Intestinal stem cell dynamics in human HSCR and control tissue.

(A to C) PHATE embedding of all stem cell marker expression (A), *CLU*, *SOX9*, *LGR5* and *OLFM4* stem cell marker expression (B), and *CLU*, *LGR5*, and *OLFM4* stem cell marker expression in the HSCR and control distal colon and rectum (C).

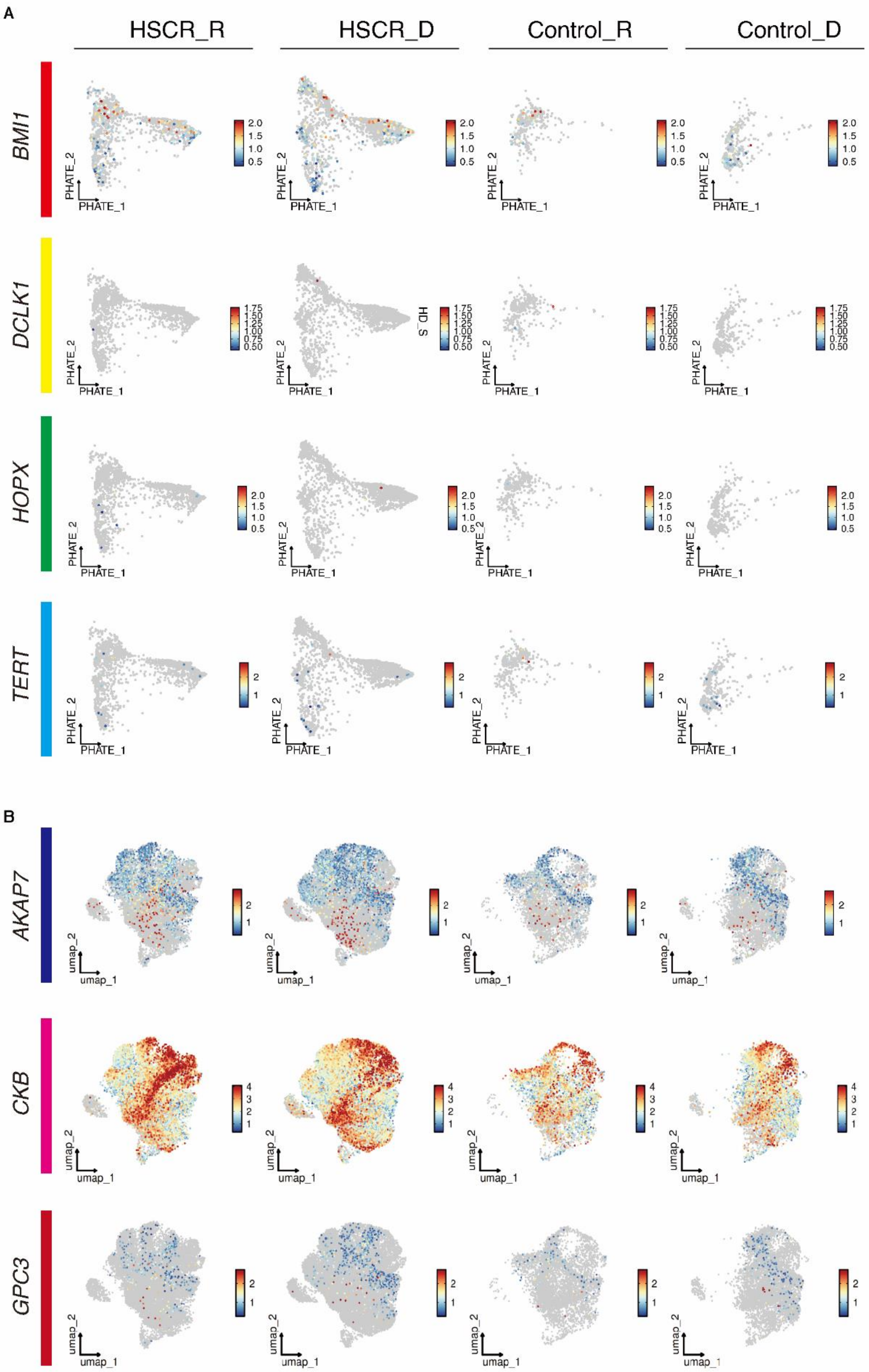
A
HSCR_R
HSCR_D
Control_R
Control_D
BMI1
DCLK1
HOPX
TERT
PHATE_1
PHATE_2
B
AKAP7
CKB
GPC3
umap_1
umap_2

Fig. S4: +4 reserve stem cells and fetal-specific distal colon epithelial progenitors in human HSCR and controls.

(A) PHATE embedding of +4 reserve stem cell (revSC) markers expression in HSCR and control distal colon and rectum. **(B)** UMAP plot visualization of fetal-specific distal colon epithelial progenitors in HSCR and control distal colon and rectum.

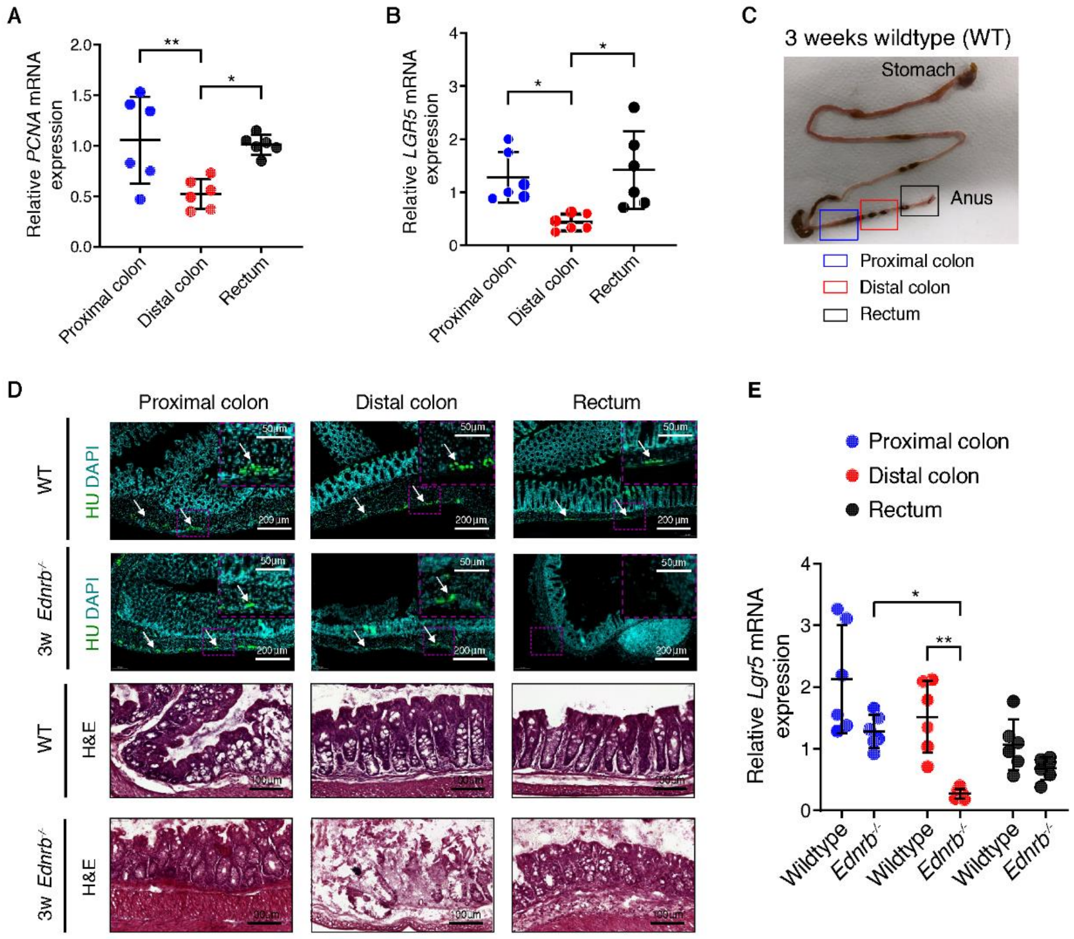

Fig. S5: mRNA expression in HAEC colons and images for 3-week-old *Ednrb*$^{-/-}$ mice.

(A and B) Relative expression of proliferation marker *PCNA* (A) and intestinal stem cell marker *LGR5* (B) mRNA in the indicated regions of human HAEC colons, normalized to the housekeeping gene *GAPDH*. **(C)** Representative image of dissected mouse whole gastrointestinal tract of wildtype (WT) 3 weeks of age. Blue box: proximal colon, red box: distal colon; and black box: rectum. **(D)** Representative immunofluorescence micrographs of neuronal marker HU (green) in the proximal colon, distal colon and rectum from 3-week-old wildtype and *Ednrb*$^{-/-}$ mice, white arrows indicate HU positive staining, with DAPI counterstaining of nuclei (blue) (scale bar = 200μm, inset = 50μm) and representative H&E-stained histomicrographs of the proximal colon, distal colon and rectum from 3-week-old wildtype and *Ednrb*$^{-/-}$ mice (scale bar = 100μm). **(E)** Relative *Lgr5* expression in the proximal colon, distal colon, and rectum from 3-week-old wildtype and *Ednrb*$^{-/-}$ mice. HAEC samples n=6. Wildtype mice n=6; *Ednrb*$^{-/-}$ mice n=6. Experiments were repeated independently 3 times, with similar results. Each dot represents the average value of each individual. Data are presented as mean ± SD and compared using one-way ANOVA with post-hoc tests. $^{*}P < 0.05$ and $^{**}P < 0.01$.

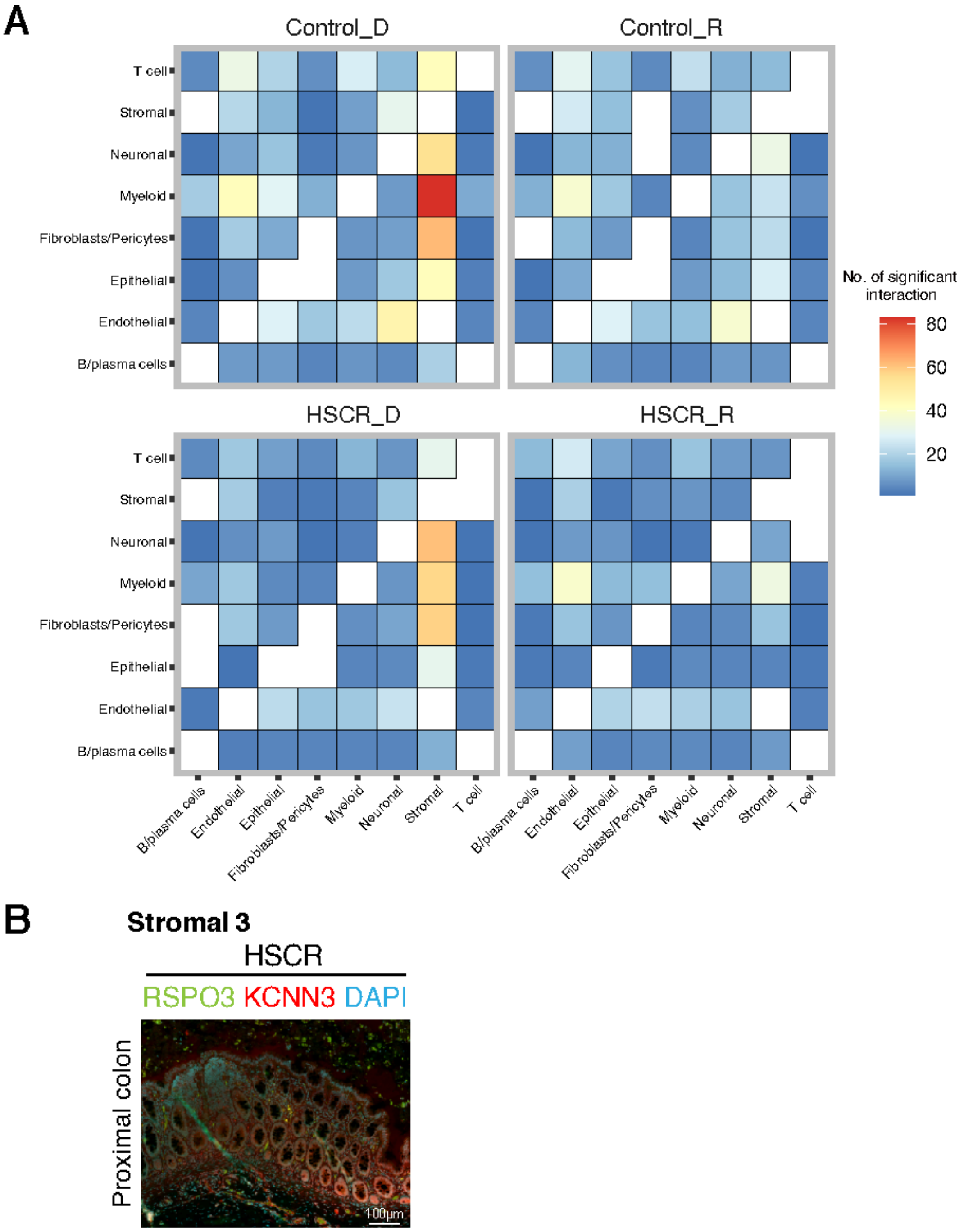

Fig. S6: Other cell types and epithelial cell interactions, and Stromal 3 and Stromal 4 cell expression in human HSCR and control tissue.

(A) Heatmaps visualizing the interaction strengths between ligands with receptors expressed by different cell types. **(B)** Representative immunofluorescence micrograph of the Stromal 3 cell markers RSPO3 (green) and KCNN3 (red) in the proximal colon of a patient with HSCR. DAPI counterstaining of nuclei (blue) (scale bar = 100µm).

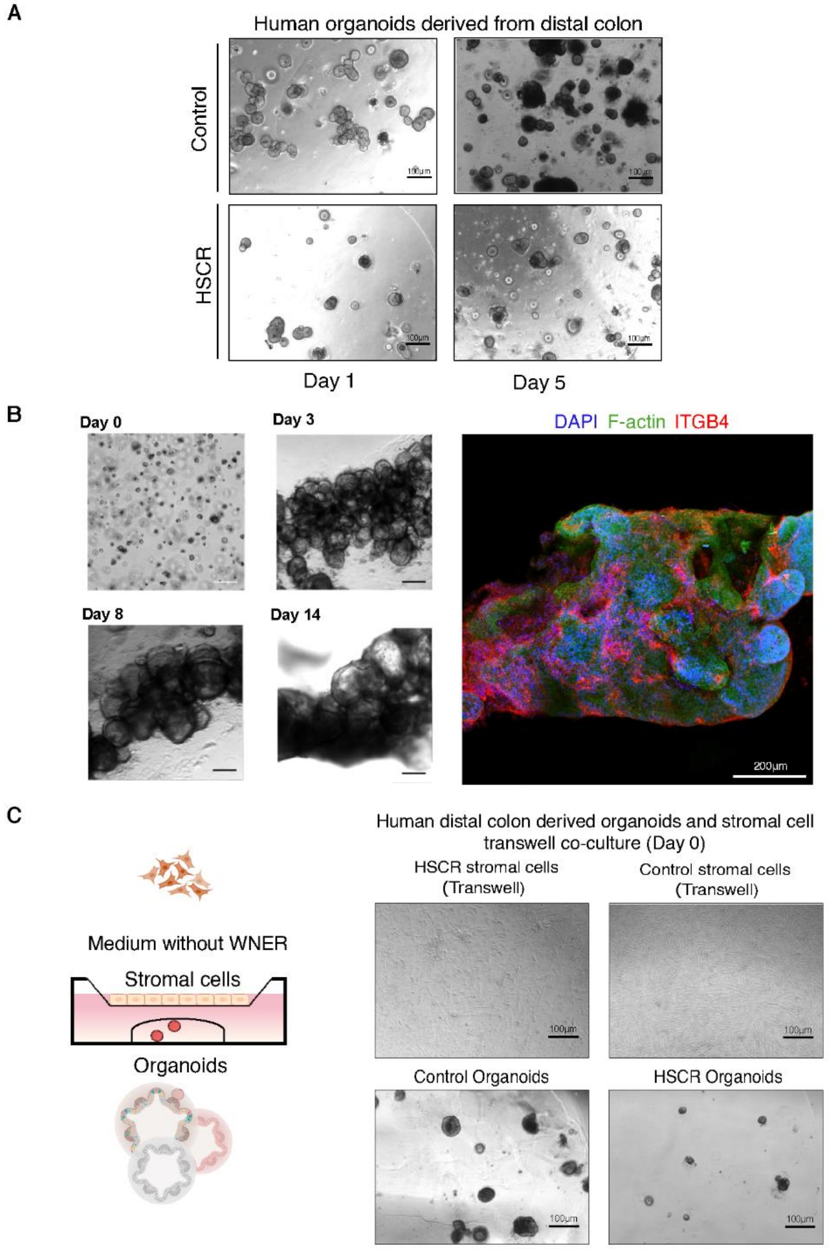

Fig. S7: Human distal colon-derived organoids and stromal cells.

(A) Representative photomicrographs of organoids derived from human HSCR and control distal colons at 1 day and 5 days in culture, respectively (scale bar = 100µm). **(B)** (Left) Representative photomicrographs of assembloid formation from HSCR distal colon-derived organoids (scale bar = 200µm) and (right) representative micrographs of immunofluorescence staining of filamentous actin (F-actin) (green) and intergrin beta 4 (ITGB4) (red), with DAPI counterstaining of nuclei

(blue) (scale bar = 200µm). **(C)** (Left) Schematic showing the co-culture model of patient tissue derived organoids together with patient tissue derived stromal cells to study the interaction between colonic epithelial cells and stromal cells in the absence of WENR growth factors. Schematic created using BioRender (https://biorender.com). (Right) Representative photomicrographs of distal colon-derived stromal cells in the transwell and distal colonic organoids in the 24-well plates before the co-cultural experiments (scale bar = 100µm). Experiments were repeated independently 3 times, with similar results.

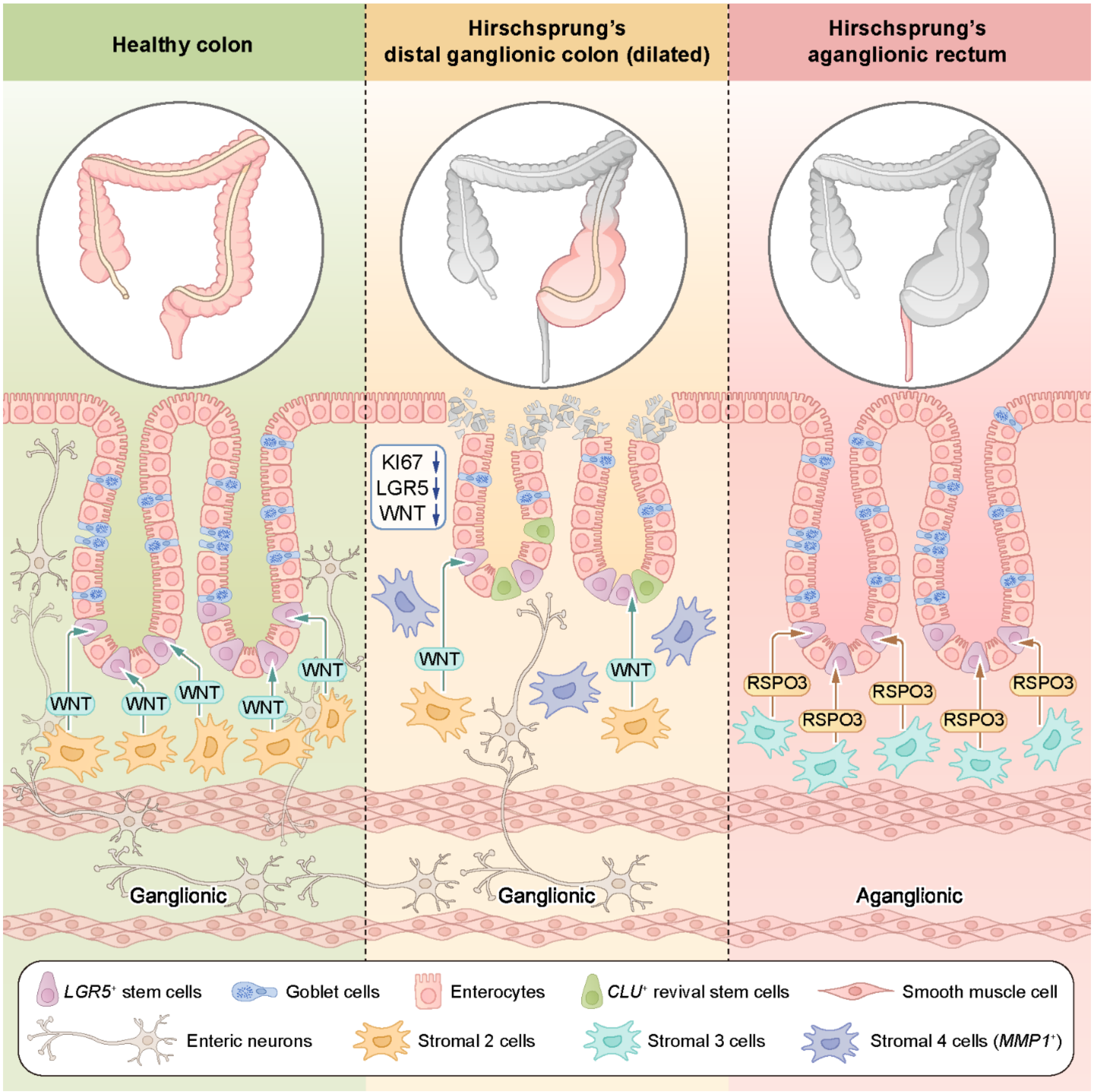

Fig. S8: Stromal remodeling and impaired epithelial regeneration in HSCR.

This schematic illustrates epithelial-stromal changes across the healthy colon (left), distal ganglionic colon in HSCR (middle), and aganglionic rectum (right). In healthy tissue, *LGR5*$^+$ stem cells are maintained by WNT-producing Stromal 2 cells, supporting epithelial regeneration. In the distal ganglionic colon of HSCR, despite preserved ganglia and no overt epithelial damage, there is reduced Wnt signaling, stemness (LGR5 ↓), and proliferation (KI67 ↓), alongside a shift from Stromal 2 to pro-inflammatory Stromal 4 (*MMP1*$^+$) cells. This precedes the onset of HAEC. In the aganglionic segment, Wnt signals are absent, and RSPO3-producing Stromal 3 cells predominate. Schematic created using BioRender (https://biorender.com).

Table S1. Further information on HSCR and control samples used in scRNA-seq analysis.

Sample_ID	Diagnosis	Segment	Age	Sex	Sample_Title	GSA_Sample Name
Subject_1	Hirschsprung disease	Distal ganglionic colon	1M7D	Male	1m HD_S	HRA004266
Subject_2	Hirschsprung disease	Aganglionic colon	1M7D	Male	1m HD_R	
Subject_3	Hirschsprung disease	Distal ganglionic colon	6M1D	Male	6m HD_S	
Subject_4	Hirschsprung disease	Aganglionic colon	6M1D	Male	6m HD_R	
Subject_5	Hirschsprung allied disease	Distal colon	1M19D	Male	1m HAD_S	
Subject_6	Hirschsprung allied disease	Rectum	1M19D	Male	1m HAD_R	
Subject_7	Hirschsprung allied disease	Distal colon	9M	Male	9m HAD_S	
Subject_8	Hirschsprung allied disease	Rectum	9M	Male	9m HAD_R	
Subject_9	Hirschsprung disease	Distal ganglionic colon	4M20D	Male	4m HD_S	
Subject_10	Hirschsprung disease	Aganglionic colon	4M20D	Male	4m HD_R	
Subject_11	Hirschsprung disease	Distal ganglionic colon	4M18D	Male	4m HD_S	
Subject_12	Hirschsprung disease	Aganglionic colon	4M18D	Male	4m HD_R	
Subject_13	Hirschsprung disease	Distal ganglionic colon	4M2D	Male	4m HD_S	
Subject_14	Hirschsprung disease	Aganglionic colon	4M2D	Male	4m HD_R	

Table S2. Gene list for Monocle2 trajectory construction.

Gene
SPINK4
AGR2
REG4
MUC1
TFF3
SPDEF
MUC2
AQP8
MS4A12
SLC26A3
CA2
CA1
KRT20
GUCA2B
CEACAM1
RPS20
RPS12
RPS19
RPS29
CFTR
CD44
PROM1
MLLT10
PTPLAD1
OLFM4
CDCA7
RGMB
PTPRO
CDK6
RNF43
DNMT3A
EZH2
STMN1
METTL3
LGR5
ASCL2

Table S3. Further information on additional HSCR, HAEC and control samples used in this study.

Sample_ID	Diagnosis	Segment	Sex	Age	Assay	Center
T_01	Hirschsprung disease	Proximal ganglionic, Distal ganglionic, Aganglionic colon	Female	4M	Histology, Immuno-staining	Toronto
T_02	Hirschsprung disease	Proximal ganglionic, Distal ganglionic, Aganglionic colon	Male	2M	Histology, Immuno-staining	Toronto
T_03	Hirschsprung disease	Proximal ganglionic, Distal ganglionic, Aganglionic colon	Male	4M	Histology, Immuno-staining	Toronto
T_04	Hirschsprung disease	Proximal ganglionic, Distal ganglionic, Aganglionic colon	Male	5M	Histology, Immuno-staining	Toronto
T_05	Hirschsprung disease	Proximal ganglionic, Distal ganglionic, Aganglionic colon	Male	6M	Histology, Immuno-staining	Toronto
T_06	Hirschsprung disease	Proximal ganglionic, Distal ganglionic, Aganglionic colon	Male	2M	Histology, Immuno-staining	Toronto
B_01	Hirschsprung-associated enterocolitis	Proximal ganglionic, Distal ganglionic, Aganglionic colon	Male	4M2D	IHC Staining, RT-qPCR	Beijing
B_02	Hirschsprung-associated enterocolitis	Proximal ganglionic, Distal ganglionic, Aganglionic colon	Male	3M12D	IHC Staining, RT-qPCR	Beijing
B_03	Hirschsprung-associated enterocolitis	Proximal ganglionic, Distal ganglionic, Aganglionic colon	Male	4M7D	IHC Staining, RT-qPCR	Beijing
B_04	Hirschsprung-associated enterocolitis	Proximal ganglionic, Distal ganglionic, Aganglionic colon	Male	3M6D	IHC Staining, RT-qPCR	Beijing
B_05	Hirschsprung-associated enterocolitis	Proximal ganglionic, Distal ganglionic,	Male	1M15D	IHC Staining, RT-qPCR	Beijing

		Aganglionic colon				
B_06	Hirschsprung-associated enterocolitis	Proximal ganglionic, Distal ganglionic, Aganglionic colon	Male	1M7D	IHC Staining, RT-qPCR	Beijing
B_07	Hirschsprung disease	Distal ganglionic colon, Aganglionic colon	Female	5M7D	IF, IHC Staining	Beijing
B_08	Hirschsprung disease	Distal ganglionic colon, Aganglionic colon	Male	6M1D	IF, IHC Staining	Beijing
B_09	Hirschsprung′s disease	Distal ganglionic colon, Aganglionic colon	Male	1M10D	IF, IHC Staining	Beijing
B_10	Anal atresia	Distal colon, Rectum	Male	1M11D	IF, IHC Staining	Beijing
B_11	Anal atresia	Distal colon, Rectum	Female	6M20D	IF, IHC Staining	Beijing
B_12	Hirschsprung′s allied disease	Distal colon, Rectum	Male	5M0D	IF, IHC Staining	Beijing
B_13	Hirschsprung disease	Distal ganglionic colon	Female	5M7D	WB, RT-qPCR	Beijing
B_14	Hirschsprung disease	Distal ganglionic colon	Male	6M1D	WB, RT-qPCR	Beijing
B_15	Hirschsprung disease	Distal ganglionic colon	Male	5M18D	WB, RT-qPCR	Beijing
B_16	Hirschsprung′s allied disease	Distal colon	Female	4M20D	WB. RT-qPCR	Beijing
B_17	Anal atresia	Distal colon	Male	6M10D	WB, RT-qPCR	Beijing
B_18	Currarino syndrome	Distal colon	Male	10M5D	WB, RT-qPCR	Beijing
B_19	Hirschsprung disease	Distal ganglionic colon	Male	4M12D	Organoids/ Stromal cells	Beijing
B_20	Hirschsprung disease	Distal ganglionic colon	Male	7M10D	Organoids/ Stromal cells	Beijing
B_21	Anal atresia	Distal colon	Male	5M18D	Organoids/ Stromal cells	Beijing
B_22	Anal atresia	Distal colon	Male	11M7D	Organoids/ Stromal cells	Beijing
L_01	Hirschsprung	Distal ganglionic	Male	22M10D	Organoids	London

	disease	colon				
L_02	Hirschsprung disease	Distal ganglionic colon	Male	22M10D	Stromal cells	London
L_03	Anorectal malformation	Distal colon	Female	9M29D	Organoids	London
L_04	Anorectal malformation	Distal colon	Female	9M29D	Stromal cells	London
B_23	Hirschsprung disease	Proximal colon, Distal colon	Male	6M7D	IF Staining	Beijing
B_24	Hirschsprung disease	Proximal colon, Distal colon	Male	6M1D	IF Staining	Beijing
B_25	Hirschsprung disease	Proximal colon, Distal colon	Male	4M20D	IF Staining	Beijing
B_26	Hirschsprung disease (with colostomy)	Proximal colon, Distal colon	Male	2M24D	IF Staining	Beijing
B_27	Hirschsprung disease (with colostomy)	Proximal colon, Distal colon	Male	2M	IF Staining	Beijing
B_28	Hirschsprung disease (with colostomy)	Proximal colon, Distal colon	Male	3M18D	IF Staining	Beijing
B_29	Hirschsprung allied disease	Proximal colon, Distal colon	Male	4M27D	IF Staining	Beijing
B_30	Hirschsprung allied disease	Proximal colon, Distal colon	Male	9M4D	IF Staining	Beijing
B_31	Hirschsprung allied disease	Proximal colon, Distal colon	Male	1M10D	IF Staining	Beijing
B_32	Hirschsprung allied disease	Proximal colon, Distal colon	Female	6M20D	IF Staining	Beijing
B_33	Hirschsprung disease	Distal colon/Proximal colon	Male	3M23D	Organoids/ Stromal cells	Beijing
B_34	Hirschsprung disease	Distal colon/Proximal colon	Male	3M27D	Organoids/ Stromal cells	Beijing
B_35	Hirschsprung disease	Distal colon/Proximal colon	Male	2M27D	Organoids/ Stromal cells	Beijing
B_36	Anal atresia	Distal colon	Female	5M23D	Stromal cells	Beijing
B_37	Anal atresia	Distal colon	Male	2M19D	Stromal cells	Beijing
B_38	Anal atresia	Distal colon	Female	2M21D	Stromal cells	Beijing
B_39	Hirschsprung	Distal	Male	2M18D	Organoids	Beijing

	disease	colon/Proximal colon				
B_40	Hirschsprung allied disease	Distal colon/Proximal colon	Female	8Y8M20D	Organoids/ Stromal cells	Beijing
B_41	Hirschsprung disease	Distal colon/Proximal colon	Male	3M12D	Organoids/ Stromal cells	Beijing
T_07	Hirschsprung disease (with colostomy)	Proximal ganglionic, Distal ganglionic, Aganglionic colon	Male	5M	Immuno-staining	Toronto
T_08	Hirschsprung disease (with colostomy)	Proximal ganglionic, Distal ganglionic, Aganglionic colon	Male	7M	Immuno-staining	Toronto
T_09	Hirschsprung disease (with colostomy)	Proximal ganglionic, Distal ganglionic, Aganglionic colon	Male	8M	Immuno-staining	Toronto
T_10	Hirschsprung disease (with colostomy)	Proximal ganglionic, Distal ganglionic, Aganglionic colon	Male	7M	Immuno-staining	Toronto
T_11	Hirschsprung disease	Proximal ganglionic, Distal ganglionic, Aganglionic colon	Male	3M	Organoids/ Stromal cells	Toronto
T_12	Hirschsprung disease	Proximal ganglionic, Distal ganglionic, Aganglionic colon	Male	2M	Organoids/ Stromal cells	Toronto

Table S4. Mouse strains used in this study.

Strain	Source	RRID
C57BL/6J	The Jackson Laboratory	RRID:IMSR_JAX:000664
B6.129P2-Lgr5tm1(cre/ERT2)Cle/J	The Jackson Laboratory	RRID:IMSR_JAX:008875
B6.129S7-Ednrb tm1Ywa/FrykJ	Juntendo University	The Hospital for Sick Children
B6.129S7-Ednrb tm1Ywa/FrykJ; B6.129P2-Lgr5tm1(cre/ERT2)Cle/J	This study	The Hospital for Sick Children

Table S5. Antibodies used in this study.

Antibody	Source	Catalogue Number and RRID
Anti-HuD + HuC antibody [EPR19098]	Abcam	Cat#: ab184267; RRID: AB_2864321
Ki67 Monoclonal Antibody (SolA15)	eBioscience	Cat#:14-5698-82; RRID: AB_10854564
MMP1 Monoclonal Antibody (6A5)	Invitrogen	Cat#: MA5-15872; RRID: AB_11154007
F3/CD142 Monoclonal Antibody (5264)	Invitrogen	Cat#: MA1-43029; RRID: AB_1087868
BMP4 Monoclonal Antibody	UltraMAB	Cat#: UM500038; RRID: AB_2629052
Human Myeloperoxidase/MPO Antibody	R&D Systems	Cat#: MAB3174; RRID: AB_2250873
Claudin 7 Polyclonal Antibody	Invitrogen	Cat#: 34-9100; RRID: AB_2533190
MUC2 Monoclonal Antibody (996/1)	Abcam	Cat#: ab11197; RRID: AB_297837
TUJ1 Antibody	BioLegend	Cat#: 801202; RRID: AB_2728521
Lgr5 Antibody	Santa Cruz	Cat#: sc68580; RRID: AB_2135160
Mucin 2/MUC2 Antibody (Ccp58)	Santa Cruz	Cat#: sc-7314; RRID: AB_627970
Anti-Ki67 Rabbit pAb	ServiceBio	Cat#: GB111499; RRID: AB_2927572
Anti-Clu Antibody	Abcam	Cat#: ab92548; AB_10585132
Goat Anti-rabbit IgG (H+L) Alexa Fluor 488 Conjugate	Cell Signaling Technology	Cat#: 4412; RRID: AB_1904025
Goat Anti-mouse IgG (H+L) Alexa Fluor 488 Conjugate	Cell Signaling Technology	Cat#: 4408; RRID: AB_10694704
Goat Anti-rat IgG (H+L) Alexa Fluor 568	Invitrogen	Cat#: A-11077; RRID: AB_2534121
Goat Anti-mouse IgG (H+L) Alexa Fluor 568	Invitrogen	Cat#: A-11004; RRID: AB_2534072
Goat Anti-rabbit IgG (H+L) Alexa Fluor 568	Invitrogen	Cat#: A-11036; RRID: AB_10563566
DAPI	Invitrogen	Cat#: D3571; RRID: AB_2307445
Horseradish enzyme labeled goat anti-mouse IgG (H+L) (affinity purification)	ZSGB-Bio	Cat#: ZB-2305; RRID: AB_2747415

Table S6. Primer sequences used for quantitative PCR.

Gene	Forward sequence	Reverse sequence
Tnfa (m)	*TTCCGAATTCACTGGAGCCTCGAA*	*TGCACCTCAGGGAAGAATCTGGAA*
Lgr5 (m)	*CGAGCCTTACAGAGCCTGATACC*	*TTGCCGTCGTCTTTATTCCATTGG*
Gapdh (m)	*TGAAGCAGGCATCTGAGGG*	*CGAAGGTGGAAGAGTGGGAG*
ASCL2 (h)	*CGTGAAGCTGGTGAACTTGG*	*GGATGTACTCCACGGCTGAG*
OLFM4 (h)	*CAGAGTGGAACGCTTGGAAT*	*CAGCTCGAAGTCCAGTTCAGT*
LGR5 (h)	*CTTCCAACCTCAGCGTCTTC*	*TTTCCCGCAAGACGTAACTC*
PCNA (h)	*GCCGAGATCTCAGCCATATT*	*ATGTACTTAGAGGTACAAAT*
AXIN2 (h)	*AAGGGCCAGGTCACCAAAC*	*CCCCCAACCCATCTTCGT*
CTNNB1 (h)	*GCAAGGCTTTTCCCAGTC*	*GAGCCCTAGTCATTGCATA*
LEF1 (h)	*GCAGCTATCAACCAGATCC*	*GATGTAGGCAGCTGTCATTC*
TCF4 (h)	*GCCTCTCATCACTACAGCA*	*GGATGGGGGATTTGTCCTAC*
WNT3a (h)	*TGTTGGGCCACAGTATTCCT*	*GGGCATGATCTCCACGTAGT*
WNT5a (h)	*GCCCAGGTTGTAATTGAAGC*	*TGGCACAGTTTCTTCTGTCC*
GAPDH (h)	*GAAGGTGAAGGTCGGAGTCAAC*	*CAGAGTTAAAAGCAGCCCTGGT*

Legends for Data files:

Data file S1. Raw data for all figures where *n*<20.

Data file S2. Differentially expressed genes for all major cell types.

Data file S3. Differentially expressed genes for epithelial cell types.

Data file S4. Differentially expressed genes for stromal cell types.

Data file S5. Differentially expressed genes along trajectories from Dynamic Time Warping analysis.

Data file S6. Drugs targeting stromal 4 from drug2cell analysis.
