# Supplementary material for: Impairment of stromal-epithelial regenerative cross-talk in Hirschsprung disease primes for the progression to enterocolitis
Source: Sci Transl Med. Author manuscript; Available in PMC 2025 Nov 10. (PMC7618341; doi:10.1126/scitranslmed.adp4679)
Supplement: Supplementary Information [file EMS210296-supplement-Supplementary_Information.pdf]

List of Supplementary Materials

Fig.S1: Epithelial cells compartment across pseudotime.

Fig. S2: Images for 2-week-old Ednrb-/- mice.

Fig. S3: Intestinal stem cell dynamic in the human HSCR and non-HSCR control tissue

Fig. S4: +4 reserve stem cells and fetal-specific distal colon epithelial progenitors in the human HSCR and non-HSCR control tissue.

Fig. S5: Gene expression in HAEC patients and images for 3-week-old Ednrb-/- mice.

Fig. S6: Other cell types and epithelial cell interaction and Stromal 3 expression in the human HSCR and non-HSCR control tissue.

Fig. S7: Human distal colon-derived organoids and stromal cells.

Table S1. Gene list for monocle2 trajectory construction.

Table S2. Differentially expressed genes for all major cell types.

Table S3. Differentially expressed genes for epithelial cell types.

Table S4. Differentially expressed genes for stromal cell types.

Table S5. Differentially expressed genes along trajectories from Dynamic Time Warping analysis.

Table S6. Drugs targeting stromal 4 from drug2cell analysis.

Table S7. Primer sequences used for quantitative PCR.

Table S8. Detailed information on Hirschsprung diseases and non-HSCR control samples used in scRNA-seq analysis.

Table S9. Detailed information on human samples used in this study.
